# Single-Cell Transcriptomic Profiles of Lung Pre-Metastatic Niche Reveal Neutrophil and Lymphatic Endothelial Cell Roles in Breast Cancer

**DOI:** 10.3390/cancers15010176

**Published:** 2022-12-28

**Authors:** Yung-Chi Huang, Chao-Yuan Chang, Yu-Yuan Wu, Kuan-Li Wu, Ying-Ming Tsai, Hsiao-Chen Lee, Eing-Mei Tsai, Ya-Ling Hsu

**Affiliations:** 1 Graduate Institute of Medicine, College of Medicine, Kaohsiung Medical University, Kaohsiung 807, Taiwan; 2 Department of Anatomy, Kaohsiung Medical University, Kaohsiung 807, Taiwan; 3 School of Medicine, College of Medicine, Kaohsiung Medical University, Kaohsiung 807, Taiwan; 4 Division of Pulmonary and Critical Care Medicine, Kaohsiung Medical University Hospital, Kaohsiung 807, Taiwan; 5 Drug Development and Value Creation Research Center, Kaohsiung Medical University, Kaohsiung 807, Taiwan; 6 Department of Biological Science and Technology, National Pingtung University of Science and Technology, Pingtung 912, Taiwan

**Keywords:** pre-metastatic niche, breast cancer, S100A6, neutrophil, lymphatic endothelial cell

## Abstract

**Simple Summary:**

The formation of a pre-metastatic niche (PMN) is important for cancer metastasis. This study revealed a specific lymphatic endothelial cell subpopulation that increased neutrophil recruitment and polarization of N2-type neutrophils in lung PMN. Our study provides an in-depth investigation for studying the characteristics of such lung PMN formation in breast cancer.

**Abstract:**

The establishment of a pre-metastatic niche (PMN) is critical for cancer metastasis. However, it remains unclear as to which phenotypes induce changes in the PMN. Single-cell transcriptomic profiling of all cells of the lung in cancer-bearing MMTV-PyVT mice revealed an increased infiltration of N2-type neutrophils and classical monocytes associated with chronic inflammation; notably, lung neutrophils isolated from mice with primary cancer exhibited similar N2-type phenotypes and expressed high levels of inflammatory and angiogenic factors. We also discovered a new cluster of Ki67-upregulated lymphatic endothelial cells (ECs) that activated several cell division-related pathways. Receptor–ligand interactions within the lung potentially mediated PMN formation; these were exemplified by the cross talk of lymphatic EC–N2-type neutrophil via S100A6. In vitro study revealed S100A6 impaired EC tight junction and increased the transendothelial migration of neutrophils. Our results highlight the molecular mechanisms that shape lung PMN and inspire preventive strategies for lung metastasis in breast cancer.

## 1. Introduction

Breast cancer (BC) is the most frequent type of human cancers and the second leading cause of cancer-related death among women worldwide [1,2]. The prognosis of patients with breast cancer, especially triple-negative breast cancer (TNBC) and human epidermal growth factor receptor 2 (HER2)-positive breast cancer, is still unsatisfactory [2,3]. In spite of the vast advancement of diagnosis and therapy in the past few decades, breast cancer patients still suffer from metastasis after diagnosis and primary tumor treatment [4], and patients are burdened by poor treatment response to the available therapeutic options [5]. It is therefore essential to conduct further in-depth research into the molecular mechanisms of breast cancer progression and metastasis.

The pre-metastatic niche (PMN) plays a crucial role in cancer development, serving as a favorable environment for colonization, survival and proliferation for disseminated cancer cells and is the rate-limiting step for cancer metastasis [6,7]. The microenvironment of metastatic target tissue undergoes a series of cellular and molecular changes to facilitate the proliferation of disseminated cancer cells [8,9]; thus, so a better understanding of PMN formation could lay the foundation for the development of metastasis preventive strategies for breast cancer metastasis. The increased recruitment of immune cells, such as macrophages, neutrophils and myeloid-derived suppressor cells (MDSCs), is a fundamental characteristic of PMNs [10,11]. In addition, it is known that vascular endothelial cells undergo angiogenesis, tight junction destruction and the secretion of inflammatory factors to support cancer cell spread [12,13]. Although the distinctive characteristics of organotropism are attributed to the complex components and precise structures between different metastatic target organs and the type of cancers [14]. Consequently, a systemic tool for investigating panoramic changes underlying PMN formation in specific organs is required.

Transcriptome analysis assessed by bulk RNA-seq is insufficient for profiling the transcriptional features in specific cell types. Recent innovations in single-cell RNA sequencing (scRNA-seq) have provided new insights for single-cell level depiction of the cellular diversity and have enabled the classification of distinct cell types in an organ [15,16], offering an unique opportunity to inspect the knowledge gap by dissecting the cellular modeling and complex interactions involved in PMN formation. In the current study, a single-cell atlas of the lung PMN from MMTV-PyVT mice was constructed, with analysis revealing that immune cells, largely driven by subtype-specific changes, were involved in PMN formation. In the midst of substantial heterogeneity, a specific lymphatic endothelial cell subpopulation was identified, marked by high S100A6 expression and contributing to neutrophil recruitment and polarization. Our study provides an in-depth investigation for studying the characteristics of lung PMN formation in breast cancer and for developing effective preventive strategies for patients with breast cancer.

## 2. Materials and Methods

### 2.1. Experimental Animals

All animals were housed in accordance with the Institutional Animal Care and Use Committee guidelines of Kaohsiung Medical University. Female and male MMTV-polyoma middle T antigen (PyMT) transgenic mice on an FVB background (double transgenic; FVB/N-Tg [MMTV-PyVT] 634 Mul∙J^−1^) [17] were purchased from Jackson Laboratories (Bar Harbor, ME, USA). The breeding program was established using FVB/NJ females and hemizygous MMTV-PyVT 634 Mul/J^−1^ male. These mice developed palpable mammary tumors at approximately 4–5 weeks, and metastases to the lung at approximately 15 weeks of age. All of the animal experiments were performed in accordance with the Institutional Animal Care and Use Committee, and with the approval of Animal Care and Use Committee of the School of Kaohsiung Medical University.

### 2.2. Lung Tissue Dissociation and CD45^+^/CD45^-^ Cell Separation

The dissociation of lung tissue was performed using a lung dissociation kit (Cat #130-095-927; Miltenyi Biotech, Bergisch Gladbach, Germany) according to the manufacturer’s instructions. Briefly, the lungs of the mice were transferred to a C tube containing the enzyme/serum free Dulbecco’s Modified Eagle Medium (DMEM) with the mix solution (enzymes D and A in DMEM medium). The GentleMACS programs were run with a 31-min incubation on the MACS mix tube rotator at 37 °C. The cell suspension was subsequently passed through a 70-µm strainer, and the CD45^+^ cells were separated using CD45 MicroBeads (Cat #130-110-618; Miltenyi Biotech; Bergisch Gladbach, Germany) in combination with LS magnetic separation columns according to the manufacturer’s instructions. CD45^-^ cells were collected in the original flow through, while CD45^+^ cells retained on the column were collected by flushing the column with buffer and removing the column from the magnetic field.

### 2.3. scRNA-seq Library Preparation and Sequencing

Immediately post cell population separation, CD45^+^ and CD45^-^ single cells were prepared for single cell sequencing using the Chromium Single Cell 3′ GEM, Library & Gel Bead kit v3 (10X Genomics, Pleasanton, CA, USA) on a 10X Genomics Chromium Controller. Generation of Gel Beads-in-Emulsion (GEM), barcoding, post GEM-reverse transcription cleanup, and amplification of the cDNA library was performed by 15 cycles of polymerase chain reaction (PCR) amplification according to the manufacturer’s instructions; the library was then sequenced using NextSeq2000 for Illumina (San Diego, CA, USA).

### 2.4. Data Quality Control, Integration and Clustering

ScRNA-seq data were processed using the Cell Ranger Single-Cell Software Suite (10X Genomics, Pleasanton, CA, USA) and then further analyzed using the R package Seurat (version 4.0.4; https://satijalab.org/seurat/, accessed on 24 December 2022) with default parameters. The data were filtered according to the following thresholds: <200 or >3000 as unique expressed genes (nFeature_RNA) and >20% as the percentage of mitochondrial genome content. The data were then normalized by converting with a scale factor (default as 10,000) and log-transformed using the Seurat embedded function. A correlation analysis was performed by employing the Run PCA function of the Seurat package, followed by an integrated analysis of the three datasets. Clustering analysis was carried out using standard Seurat package procedures with a resolution at 1.2. The identified clusters were then visualized using Uniform Manifold Approximation and Projection (UMAP) of the principal components in Seurat.

### 2.5. Bioinformatic Analysis of Differential Expression Analysis and Trajectory Analysis

Signature genes were required to be expressed in >50% of cells within either of the two cell groups and the changes in gene expression were >2-fold (log_2_FC  > 1). Pathway enrichment analysis examining the enriched processes in clusters was performed using Ingenuity Pathway Analysis (IPA, Qiagen, Hilden, Germany) and Kyoto Encyclopedia of Genes and Genomes (KEGG) pathway analysis. To uncover the pathway that is potentially linked to the identified modules of analysis, trajectory analysis was performed by Monocle 2 (v2.22) [18,19].

### 2.6. Weighted Gene Co-Expression Network Analysis (WGCNA)

The accessible R package of hdWGCNA (v0.1.1.9002) for scRNA-seq was used for co-expression analysis (https://github.com/smorabit/hdWGCNA, accessed on 9 June 2022) [20]. According to the manufacturer’s instructions, one-step network construction and module detection was used; the module eigengene expression, adjacency matrix heat-map and other related parameters/results were calculated and visualized.

### 2.7. Cell–Cell Communication

A database of known cytokine/chemokine receptor and ligand pairs was curated using the combined information from CellTalkDB [21] (http://tcm.zju.edu.cn/celltalkdb/, accessed on 25 February 2022), CellPhoneDB [22] (https://www.cellphonedb.org/, accessed on 20 March 2022), CellChat (v1.1.1) (https://github.com/sqjin/CellChat, accessed on 20 June 2022) [23], and resources from previously published literature. This database was then filtered for possible receptor–ligand interactions using the following criteria: (1) each receptor or ligand should be expressed in at least 50% of the cells of an individual cluster; (2) each pair should be expressed within the secretory and recipient cells.

### 2.8. Neutrophil Isolation, Phenotype Analysis and Secretome Analysis

Murine neutrophils were isolated from the lungs of wild-type or MMTV-PyVT mice at 12 weeks of age using the Mouse Neutrophil Isolation kit (Miltenyi Biotech) after lung digestion as described above. The markers and arginase I were assessed using flow cytometry after specific antibody staining. The antibodies used in this study are listed in Table S29. Fresh isolated lung neutrophils from mice were cultured for 48 h and the supernatants of the neutrophils were then assessed using the Luminex (R&D system, Minneapolis, MN, USA) and qRT-PCR. The primers used in this study are listed in Table S28.

### 2.9. Permeability and Transendothelial Migration

Human microvascular endothelial HULEC-5a cells (ATCC, CRL-3244) were cultured in MCDB131 medium containing 10% FBS, 10 ng/mL epidermal growth factor (EGF), 1 µg/mL hydrocortisone and 10 mM glutamine (ThermoFisher Scientific, Waltham, MA, USA). The permeability of HULEC-5a cells was assessed using an in vitro Vascular Permeability Assay kit (EMD Millipore, Burlington, MA, USA). Briefly, HULEC-5a cells were seeded and then treated with various concentrations of S100A6 protein (R&D system) for 3 days to confluence in inserts. FITC-Dextran solution was then added to the monolayer of HULEC-5a cells and incubated for 1 h. The medium in the bottom well was collected and the FITC-Dextran was measured (excitation at 485 nm, emission at 535 nm). HULEC-5a cells were seeded and grown to confluence in an insert (pore size: 1 μm, EMD Millipore, Burlington, MA, USA). Freshly isolated human neutrophils (5 × 10^5^ cells) were added to the inserts, and coculture was carried out for 12 h. The migratory neutrophils in the bottom well were then counted.

### 2.10. CODEX Staining and Imaging of the Lungs of Mice

Barcode-conjugated antibody staining of tissue sections mounted on poly-lysine coated cover slips was performed using a commercially available CODEX Staining kit [24] for FFPE tissue using a commercial Akoya CODEX instrument (Akoya Biosciences, Marlborough, MA, USA) with a Keyence BZ-X800 microscope plus Nikon PlanApo NA 0.75 objective. Images were acquired at 20X magnification and filter cubes for TRITC (550), CY5 (647), and CY7 (750) complementary fluorescent reporters and DAPI (Akoya Biosciences). Typical images are 3 × 3 mm including acquisition of 5 Z-stack images. The images of specific cell type and the quantitation were collected using the CODEX Processor software (version 1.30.0.12, Akoya Biosciences, Menlo Park, CA, USA). The antibodies used in this study are listed in Table S6.

### 2.11. Statistical Analysis

Statistical analysis was performed using Prism version 9.2 (GraphPad Software, San Diego, CA, USA). One-way analysis of variance was used to identify differences between two or more experimental groups, and Student’s unpaired *t*-tests were used where comparisons were made between two treatment groups. All results are presented as mean ± standard error of the mean (SEM), and *p* values of <0.05 were considered statistically significant.

## 3. Results

### 3.1. Single-Cell Transcriptome Atlas in the Lung Pre-Metastatic Niche of Breast Cancer

**Figure 1:**
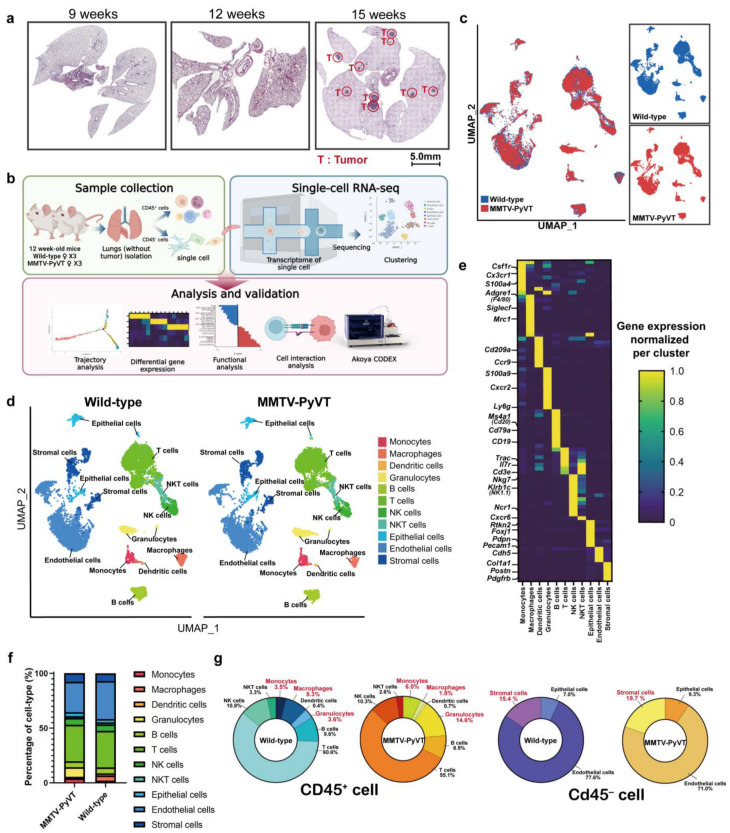
Transcriptome atlas of the lung pre-metastatic niche in MMTV-PyVT mice with primary breast cancer. (**a**) HE staining of lungs in wild-type and MMTV-PyVT mice. The lungs were collected from wild-type and MMTV-PyVT mice with primary breast cancer at 9, 12 and 15 weeks of age (*n* = 3). (**b**) Experimental flowchart. (**c**) Blueprint of the scRNA-seq of the lung. (**d**) Cell clusters of the lungs of wild-type and MMTV-PyVT mice. UMAP representation of the lungs of wild-type and MMTV-PyVT mice. (**e**) Markers of cell clusters. (**f**) Cell number of each cell cluster. (**g**) Pie chart for the percentage of Cd45^+^ and Cd45^-^ cells.

In order to create a comprehensive cellular map of normal and lung PMN in breast cancer, scRNA-seq analysis was implemented on the lung from wild-type and MMTV-PyVT mice at 12 weeks, when there were no obvious tumor nodules in the lungs (Figure 1a). We also isolated populations of Cd45^+^ (immune cells) and Cd45^-^ (non-immune cells) cells to capture the diverse cell populations presented in the lung (Figure 1b). All scRNA-seq data were merged, normalized, batch-effect corrected and clustered to identify the cell types (Figure S1a–c). The transcriptomes of 12,684 or 9965 cells obtained from three fresh lungs of wild-type or MMTV-PyVT mice were profiled respectively then cells were clustered according to the Uniform Manifold Approximation and Projection (UMAP) analysis, and these clusters were annotated based on established cell markers (Table S1). A total of 11 clusters were annotated corresponding to five main cell populations: myeloid cells (monocytes, macrophages, dendritic cells (DCs) and granulocytes), lymphoid cells (B cells, T cells, natural killer (NK) cells and NKT cells), epithelial cells (Type I and Type II alveolar epithelial cells, club cells and ciliated cells), endothelial cells (ECs) and stromal cells (fibroblasts, fibromo/SMCs, mesothelial cells and satellite glial cells) (Figure 1c–e). It was found that the numbers of granulocytes, monocytes and stromal cells were increased in the lungs of MMTV-PyVT mice (Figure 1f,g).

### 3.2. Breast Cancer Alters the Immune Ecosystem of the Lung

**Figure 2:**
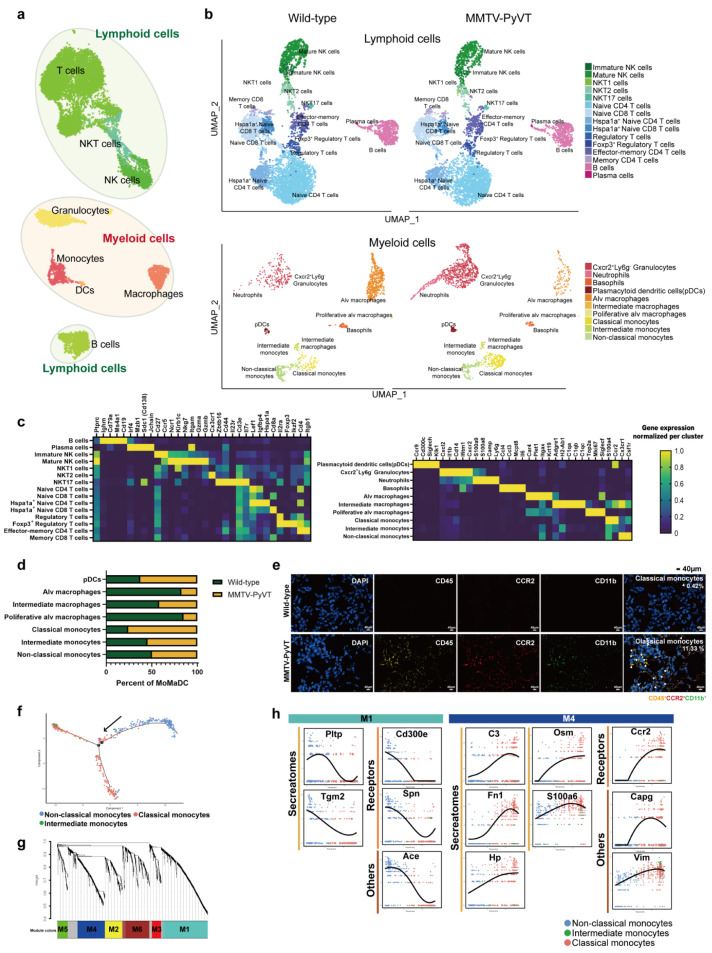
Atlas of immune cells. (**a**) Blueprint of populations of lymphoid and myeloid cells. (**b**) Immune cell (Cd45^+^ cells) clusters from 10X Genomics scRNA-seq analysis visualized by UMAP. (**c**) The specific biomarkers of each cell cluster. (**d**) The cell number of monocytes/macrophages/DCs. (**e**) Increased cell number was found in the lungs of MMTV-PyVT mice by CODEX Multiplexed Tissue Staining. The lungs of wild-type and MMTV-PyVT mice were collected and stained by CODEX Multiplexed Tissue Staining. Classical monocytes (CD45^+^ (yellow) CCR2^+^ (red) CD11b^+^ (green) cells) are indicated by white arrows. The percentage of classical monocytes was indicated as a percentage of total CD45^+^ cells. Scale bars, 40 μm. (**f**) Trajectory analysis of monocytes. The trajectory analysis of single-cell data was performed using the Monocle 2 algorithm. (**g**) Analysis of WGCNA. The sequence diagram is shown at the top of the image. The bottom of the figure showed the different color gene modules and the number of bases they contained. (**h**) Genes contributing to the functional differences in non-classical and classical monocytes.

Analysis of CD45^+^ immune cells from the lungs of wild-type and MMTV-PyVT mice with SingleR revealed eight distinct clusters with specific markers (Figure 2a). They were defined as immune cell types, including B cells, DCs, granulocytes, macrophages, monocytes, NK cells, NKT cells and T cells. The expression of classical markers for lymphoid cells (*Cd79a* for B cells, *Cd3e* for T cells, *Ncr1* for NK cells and *Cd3e* plus *Klrb1c* for NKT cells), monocytes (*Csf1r*), macrophages (*Mrc1*), DCs (*Cd209a*) and granulocytes (*Cxcr2*) are shown in the corresponding cell clusters (Figure 2b). By examining these cells in detail, 25 sub-clusters were annotated (Figure 2b,c), and to better understand the involvement of monocytes/macrophages/DCs in the formation of lung PMNs, we classified seven subsets of these myeloid cells, including plasmacytoid dendritic cells (pDCs), alveolar macrophages (alv mø), intermediate mø, proliferative mø, classical monocytes, intermediate monocytes and non-classical monocytes (Figure S2a,b). The cell number of classical monocytes was markedly increased whereas alv mø and proliferative mø were decreased in the lungs of MMTV-PyVT mice (Figure 2d). The increase in classical monocytes was also validated by a CODEX-based staining (Figure 2e). Trajectory analysis showed a gradual transition from the root non-classical monocytes state to the intermediate monocytes and then the classical monocytes (Figure 2f and Table S2). WGCNA analysis, a method to identify networks of connecting genes from transcriptomes [25], showed that M1 and M2 modules were positively correlated with non-classical monocytes, whereas M4 module was strongly correlated with classical monocytes (Figure 2g, Figures S3a–d and S4; Table S3). The gene set of M4 module underwent KEGG analysis and revealed association with “immune response trigger” (Figure 2g and Figure S5a,b; Tables S4 and S5). To investigate hub genes contributing to non-classical and classical monocyte transition, we intersected the genes of trajectory analysis and WGCNA (M1 and M4). The results revealed that *Pltp*, *Tgm2*, *Cd300e*, *Spn* and *Ace* dropped markedly in cells as they transformed from non-classical monocytes into classical monocytes, while *C3*, *Fn1*, *Hp*, *Osm*, *S100a6*, *Ccr2*, *Capg* and *Vim*, were upregulated in cells as they transformed into classical monocytes (Figure 2h and Figure S6a; Table S3). A comparison of the differentially expressed (DE) genes in classical monocytes from the lungs of wild-type and MMTV-PyVT mice revealed that the expression of inflammatory genes *S100a9*, *Slpi*, *Wfdc17* and *Apoc2* was upregulated (log2(FC) > 1, expression in >50% cells) in classical monocytes of the lungs of wild-type mice, whereas the expression of *Irf8* and *Jun* was downregulated (−log2(FC) < 1, expression in >50% cells) (Figure S6b).

**Figure 3:**
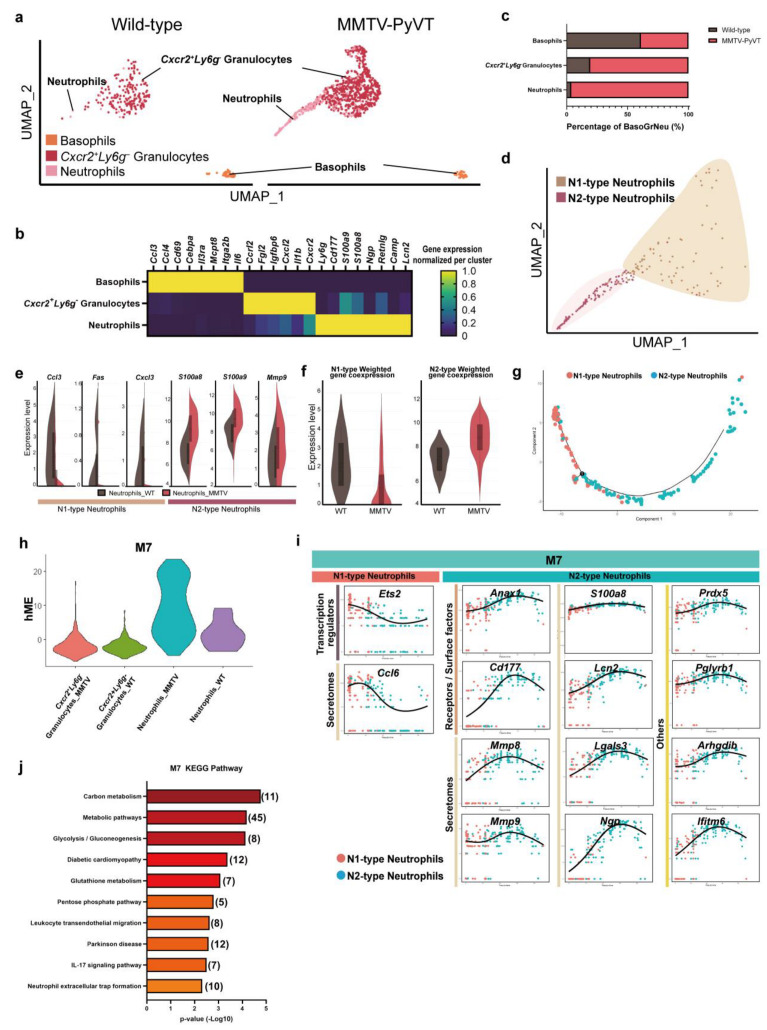
N2-type neutrophils identified in lung PMN. (**a**) Subpopulation of granulocytes. (**b**) Biomarkers of granulocyte subpopulations. (**c**) The cell number for granulocyte subpopulations. (**d**) UMAP analysis representing the distribution of N1- and N2-type neutrophils. (**e**) Violin plot showing the expression of N1 and N2 markers in the neutrophils of wild-type and MMTV-PyVT mice. (**f**) Weighted analysis of neutrophil phenotypes of wild-type and MMTV-PyVT mice. (**g**) Trajectory analysis of N1- and N2-type neutrophils. (**h**) WGCNA analysis of neutrophils in the lungs of wild-type and MMTV-PyVT mice. Each leaf (vertical line) in the dendrogram corresponded to a gene. (**i**) KEGG pathway analysis of M4 module of the WGCNA analysis. (**j**) Genes that contributed to the functional transition of N1- and N2-type neutrophils.

We next focused on the granulocytes and further characterized their potential biological states. Following analysis of the granulocytes, we identified three distinct clusters, basophils, neutrophils (*Cxcr2^+^Ly6g^+^*) and *Cxcr2^+^Ly6g^-^* granulocytes with specific markers (Figure 3a,b). The number of *Cxcr2^+^Ly6g^-^* granulocytes and neutrophils was dramatically increased in the lungs of MMTV-PyVT mice (Figure 3c). Trajectory analysis showed that they began as *Cxcr2^+^Ly6g^-^* granulocytes, and then progressed to a neutrophil population followed by basophils (Figure S7a).

Previous studies indicated that neutrophil polarization influences the role they play in the tumor microenvironment [26], so we then determined the phenotypes of the neutrophils, with results indicating that markers of N2-type neutrophils, such as *Mmp9* and *S100a8/9* were increased, whereas N1 markers *Ccl3*, *Fas* and *Cxcl3* were decreased in the neutrophils of MMTV-PyVT mice (Figure 3d,e). WGCNA also showed that neutrophils from the lungs of MMTV-PyVT mice exhibited a N2-type phenotype (Figure 3f). Screening was performed for genes whose expression levels were correlated with N1- or N2-type by N1–N2-type trajectory analysis. The analysis yielded 28 genes whose increased expression levels were associated with N1-type and 25 genes whose expression levels were associated with N2-type (Figure 3g and Figure S7b; Table S6). In addition, WGCNA analysis identified 10 modules of interest; M7 module had the most significant difference on the transition of N1 to N2-type neutrophils (Figure 3h, Figures S8a–g and S9; Tables S7–S12). After analyzing the intersection of these two analysis databases, 14 genes were found to be involved in the phenotypic switch between N1/N2 neutrophils. Our analysis revealed an increased expression of transcription factor *Ets2* and cytokine *Ccl6* associated with the N1 phenotype, whereas increased expression of receptors (*Anxa1* and *Cd177*) and secretory factors (*Mmp8*, *Mmp9*, *S100a8*, *Lcn2*, *Lgals3*, *Ngp*, *Prdx5*, *Pglyrb1*, *Arhgdib* and *Ifitm6*) were associated with the N2-type phenotype (Figure 3i). Pathway analysis of M7 module showed that metabolism, leukocyte migration and NET cascade were involved in the phenotypic transition of neutrophils (Figure 3j and Figure S10a–d).

### 3.3. Breast Cancer Increased the Infiltration and Polarization of N2-Type Neutrophils in the Lung PMN in MMTV-PyVT Mice

**Figure 4:**
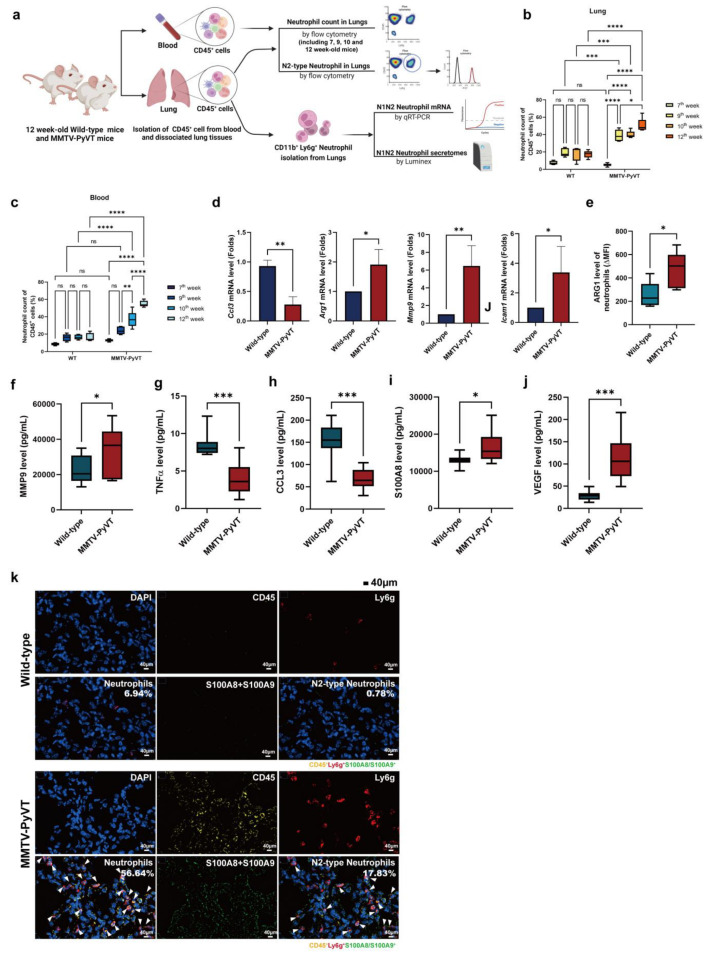
The infiltration and phenotypes of neutrophils in the blood and lungs of MMTV-PyVT mice. (**a**) The workflow of blood and lung neutrophil isolation. (**b**) The cell number of neutrophils in the lungs of wild-type and MMTV-PyVT mice. (**c**) The cell number of neutrophils in the blood of wild-type and MMTV-PyVT mice. (**d**) Expression of *Arg1*, *Icam1*, *Mmp9* and *Ccl3* in neutrophils, as determined by qRT-PCR. The protein levels of (**e**) ARG, (**f**) MMP9, (**g**) TNFα, (**h**) CCL3, (**i**) S100A8 and (**j**) VEGF in neutrophils of the lungs of mice (*n* = 7–11). The expression of arginase I on fresh isolated neutrophils was assessed by flow cytometry. The levels of various soluble factors in the supernatant were detected by a Luminex system after 24-h culture. * *p*  <  0.05, ** *p*  <  0.01, *** *p*  <  0.001, **** *p*  <  0.0001. (**k**) N2-type neutrophils were found in the lungs of MMTV-PyVT mice. The lungs of wild-type and MMTV-PyVT mice was collected and stained by *CODEX-based* multiplexed tissue *staining*. N2-type neutrophils (CD45^+^ (yellow) Ly6G^+^ (red) S100A8/A9^+^ (green) cells) were indicated by the white arrow. The percentage of N2-type neutrophil was indicated as a percentage of total CD45^+^ cells. Scale bars, 40 μm.

To determine whether N2-type neutrophils were recruited to the lung PMN in vivo, we isolated neutrophils from the blood and lungs of wild-type and MMTV-PyVT mice (Figure 4a). As shown in Figure 4b,c, the data showed the cell number of neutrophils was no significant difference in both blood and lung at 7, 9, 10 and 12 weeks of age in wild-type mice. The cell number of neutrophils showed no difference between the lungs of wild-type and MMTV-PyVT mice at 7 weeks of age, suggesting that changes of neutrophils was not due to PyVT oncoprotein non-specific expression. However, the cell number of neutrophils increased in the blood and lungs of MMTV-PyVT mice at 9 to 12 weeks of age (Figure 4c). Quantitative reverse transcription-polymerase chain reaction (qRT-PCR) revealed that expression of the N1 marker *Ccl3* was decreased, whereas *Arg1*, *Mmp9* and *Icam1* were increased in neutrophils from the lungs of MMTV-PyVT mice (Figure 4d). Phenotypic analysis of surface markers and the secretomes also revealed that infiltrated neutrophils expressed the upregulated N2 markers arginase I (ARG1) and MMP9, whereas the N1 markers TNFα and CCL3 were downregulated in the lungs of MMTV-PyVT mice (Figure 4e–h). In addition, inflammatory and angiogenic factors, including S100A8 and VEGF were also increased in the lungs of cancer-bearing MMTV-PyVT mice, compared with wild-type mice (Figure 4i,j). CODEX-based staining also showed that neutrophils (CD45^+^Ly6g^+^) expressed S100A8/A9 (M7 module) proteins (Figure 4k). Collectively, these data suggest that N2-type neutrophils predominantly accumulate in the lung PMN in MMTV-PyVT mice.

### 3.4. Breast Cancer Alters the Transcriptome of ECs

**Figure 5:**
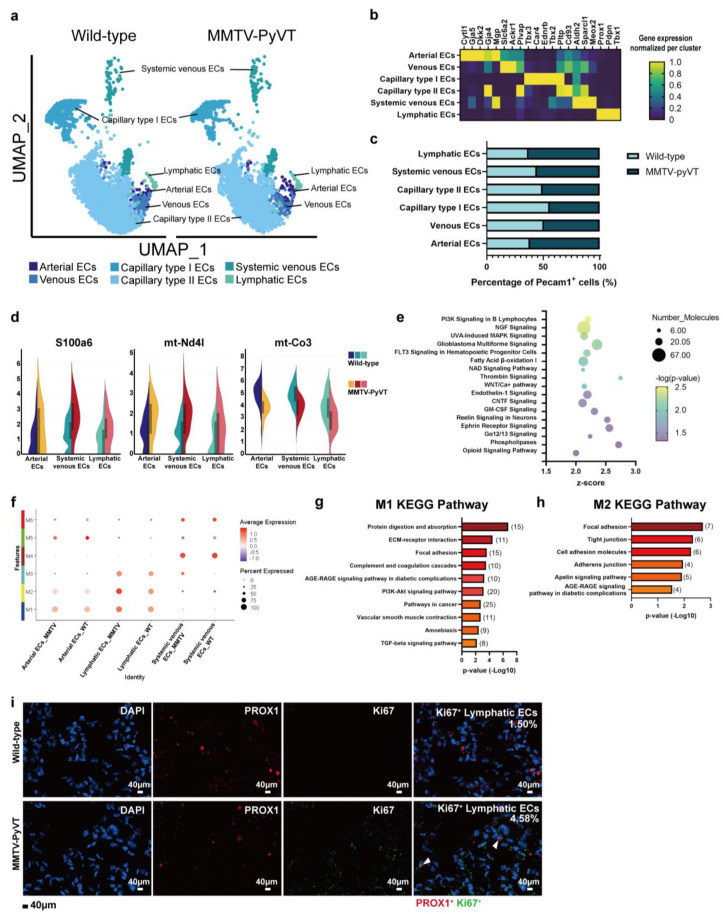
Detection of endothelial cell clusters in lungs of MMTV-PyVT mice. (**a**) Subpopulations of ECs in the lungs of mice. (**b**) Biomarkers for the subpopulations of ECs. (**c**) The cell numbers for each subpopulation of ECs. (**d**) Violin plots of differentially *expressed genes* in all EC subpopulations in wild-type and MMTV-PVT mice. (**e**) Pathway analysis of the transcriptome of lymphatic ECs in the lung. (**f**) WGCNA analysis of ECs in wild-type and MMTV-PyVT mice. The KEGG pathways of M1 (**g**) and M2 (**h**) modules of WGCNA. (**i**) Proliferative lymphatic ECs were found in the lungs of MMTV-PyVT mice. Tissue samples of the lungs of wild-type and MMTV-PyVT mice were collected and stained by *CODEX* Multiplexed Tissue *Staining*. Proliferative lymphatic ECs (PROX1^+^ (red) Ki67^+^ (green)) were indicated by white arrow. The percentage of proliferative lymphatic ECs were indicated as a percentage of total CD31^+^ cells. Scale bars, 40 μm.

By analyzing ECs (*Pecam1^+^Cdh5^+^*), we identified six distinct clusters: arterial ECs, venous ECs, capillary type I (aerocytes), capillary type II, systemic venous and lymphatic ECs (Figure 5a,b). Trajectory analysis revealed that the root was mainly populated by capillary type I ECs, while the terminus of the three ECs was populated by lymphatic ECs (Figure S11a). The cell number of arterial, lymphatic and systemic venous ECs was increased, whereas capillary type I (aerocytes) showed slight decrease in the lungs of MMVT-PyVT mice. The cell number of capillary type II ECs was not changed in the lung PMN of mice (Figure 5c). A Venn diagram of the DE genes in altered EC clusters revealed that two factors (*S100a6* and *mt-Nd4l*) were upregulated with one gene (*mt-Co3*) but downregulated in the ECs of lungs of MMTV-PyVT mice (Figure 5d and Figure S11b).

The changes in the number of lymphatic ECs in the lungs of MMTV-PyVT mice were determined with IPA analysis showing that 10 pathways were activated (Z-score > 2, *p* value < 0.05), including “PI3K signaling”, “NGF signaling”, “UVA-induced MAPK signaling”, “GBM signaling”, “FLT3 signaling”, “Fatty acid β-oxidation”, “NDA signaling pathway”, “Thrombin signaling”, “Wnt/calcium” and the “Endothelin-1 pathway” (Figure 5e and Table S13). Nras and Pik3ca were involved in all signaling pathways (Figure S11c). Moreover, WGCNA revealed that M1 and M2 modules were positively correlated with lymphatic ECs and also contributed to changes in lymphatic ECs between wild-type and MMTV-PyVT mice (Figure 5f, Figures S12a–c and S13; Table S14). The KEGG pathway of hub genes of M1 module included “Protein digestion and absorption”, “ECM receptor interaction”, “Focal adhesion”, “Complement and coagulation”, “AGE-RAGE pathway”, “PI3K signaling” and “pathway in cancer” (Figure 5g and Table S15), while “Focal adhesion”, “Tight junction”, “Cell adhesion molecules”, “Adhesion junction” and “AGE-RAGE” pathways were involved in M2 module (Figure 5h and Table S16). CODEX-based staining also showed that there was an increase in proliferative lymphatic ECs (PROX1^+^Ki67^+^) in the lungs of MMTV-PyVT mice (Figure 5i).

### 3.5. Breast Cancer Induces Remodeling in Murine Lung Stroma

**Figure 6:**
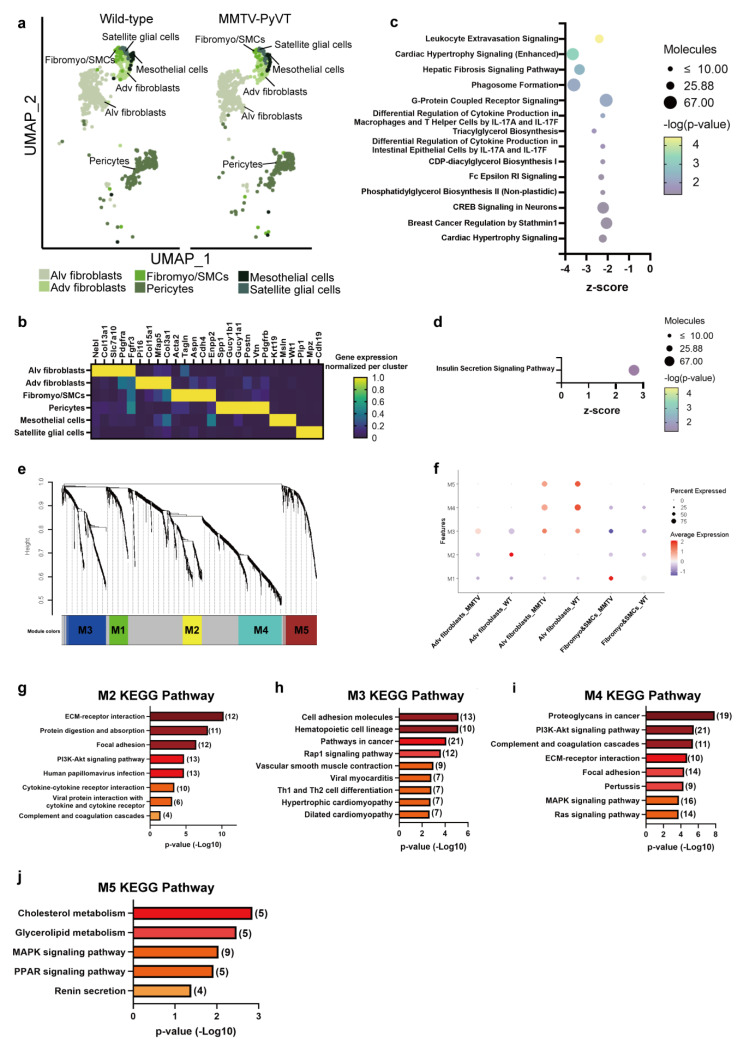
Detection of stromal clusters in the lungs of MMTV-PyVT mice. (**a**) Subpopulation of stromal cells (fibroblasts, pericytes, mesothelial cells and satellite glial cells) in the lungs of wild-type and MMTV-PyVT mice. (**b**) Biomarkers of stromal cells. Pathway analysis of (**c**) alv fibroblasts and (**d**) adv fibroblasts. (**e**) WGCNA of lung fibroblasts. (**f**) Correlation analysis between WGCNA modules and the subpopulations of lung fibroblasts. KEGG pathways (**g**) M2, (**h**) M3, (**i**) M4 and (**j**) M5 modules.

To better understand the remodeling of stroma during lung PMN formation, we first identified four clusters of lung stroma including fibroblasts/smooth muscle cells, pericytes, mesothelial cells and satellite glial cells (Figure 6a). Three clusters of fibroblasts were annotated as adventitia (adv) fibroblast, alveoli (alv) fibroblasts and fibromyo/SMCs (Figure 6a,b). To gain further insights into the potential functions of these distinct fibroblast subsets, we compared their gene expression profiles between fibroblasts of lungs of wild-type and MMTV-PyVT mice, with IPA analysis revealing that 14 pathways were inhibited in alv fibroblasts from the lungs of MMTV-PyVT mice, and the top 5 pathways were “Leukocyte extravasation”, “Cardiac hypertrophy signaling”, “Hepatic Fibrosis signaling, “Phagosome formation” and “G-protein couple receptor signaling” (Figure 6c and Table S17). Only the “Insulin secretion signaling pathway” was activated in the adv fibroblasts in the lungs of MMTV-PyVT mice (Figure 6d and Table S18). WGCNA analysis showed that M2 and M3 modules were negatively and positively associated with adv fibroblasts from the lungs of MMTV-PyVT mice, respectively (Table S19). M4 and M5 Modules were negatively associated with alv fibroblasts, whereas M1 module was positively associated with myofibroblasts of the lungs of MMTV-PyVT mice (Figure 6e,f). The eigengene of module expression across all single cells is presented in Figure S14a–e. The top 5 pathways of M2 module were “ECM-receptor interaction”, “Protein digestion and absorption”, “Focal adherence”, “PI3K/AKT pathway” and “Cytokines-cytokines interaction”. In M3 module, “Cell adhesion molecules”, “Hematopoietic cell lineage”, “Pathway in cancer” and “Rap1 signaling pathway” were the major signaling cascades, “Proteoglycans in cancer”, “PI3K/AKT signaling pathway”, “Complement coagulation cascade” and “ECM-receptor interaction” were involved in M4 module, while “Cholesterol metabolism”, “Glycolipid metabolism”, “MAPK signaling pathway” and “PPAR signaling pathway” were the main pathways in M5 module (Figure 6g–j and Figure S15; Table S20–S23).

### 3.6. Multifaceted Cell–Cell Communication Networks in the Lung PMN of Mice

**Figure 7:**
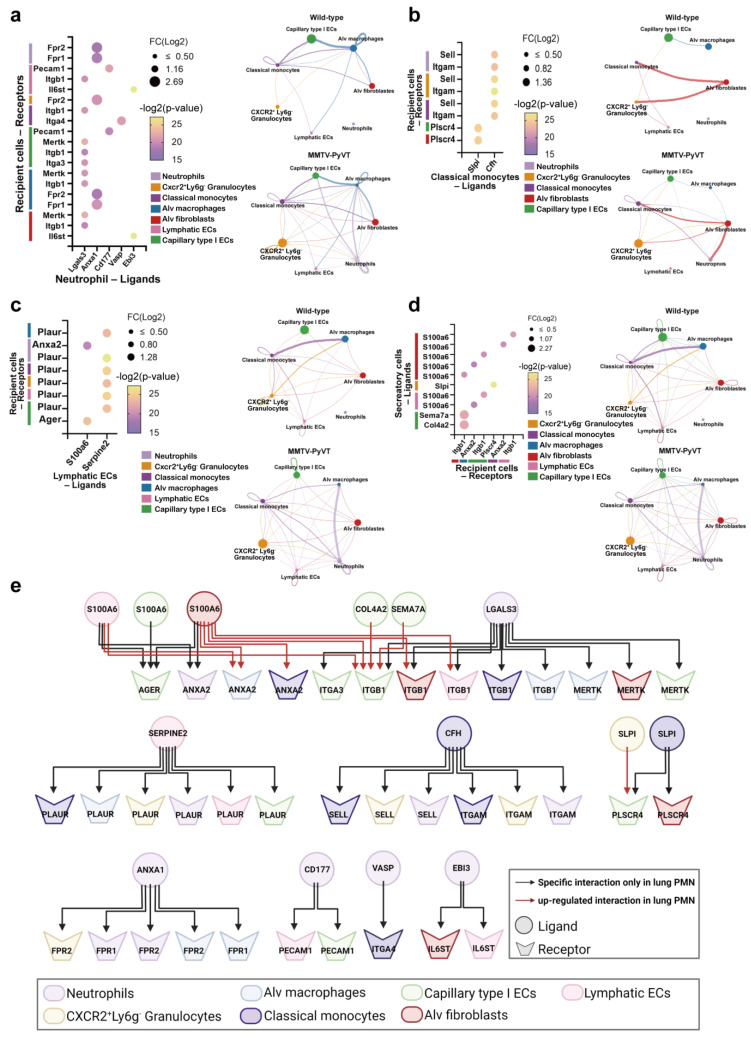
Interaction of cell types in the lungs of MMTV-PyVT mice. (**a**) The interaction of neutrophils with other cell types in the lungs of MMTV-PyVT mice. (**b**) Classical monocytes acted as secretary cells in the lungs of MMTV-PyVT mice. (**c**) Elevated S100A6-mediated interactions of lymphatic ECs in MMTV-PyVT mice. (**d**) S100A6 contributed to the interactions between alv fibroblast with other type cells. (**e**) Integrated critical network of cell–cell interactions in the lung PMN of MMTV-PyVT mice.

Our single-cell characterization of the lung PMN also allowed us to investigate potential interactions between cell compartments of the lung that may help to shape the lung PMN of breast cancer. We assessed the DE ligand–receptor pairs within subtypes, focusing on cell–cell interactions only presented in the lungs of MMTV-PyVT mice. Increased cross talk of neutrophils with other immune cells in the lung PMN of MMTV-PyVT mice was found, including with classical monocytes, alv macrophages and granulocytes, particularly via the *Anxa1*-*Fpr1* axis. In addition, there was a relatively high level of interaction between neutrophils and lung stromal cells, including capillary type I ECs, lymphatic ECs and alv fibroblasts, by *Lgals3*, where integrin (α3 and β1) and *Mertk* molecules were expressed in the respective cells (Figure 7a and Table S24).

Similar to the potential cross talk between neutrophils and other immune cells, we identified possible interactions between classical monocyte-neutrophils/granulocytes by *Cfh*-*Itgam* or classical monocyte-capillary type I ECs/alv fibroblasts by the *Spli*-*plscr4* axis in the lungs of MMTV-PyVT mice (Figure 7b). Lymphatic ECs also participated in the modification of immune cells, and consistent with the aforementioned data, computational analysis revealed that lymphatic ECs interacted with neutrophils via *S100a6* through the *Anxa2* axis in the lung PMN of MMTV-PyVT mice. The *Serpine2*-*Pleur* axis was also involved in the communication of lymphatic ECs with *Cxcr2^+^Ly6g^-^* granulocytes, alv macrophages and classical monocytes (Figure 7c) in the lung PMN of breast cancer.

We also found relatively high levels of interactions in the lungs of MMTV mice, compared with that of wild-type mice. Interestingly, *S100a6* was the main mediator contributing to the interaction of lymphatic ECs and alv macrophages with various recipient cells, including capillary type I ECs and classical monocytes (Figure 7d and Table S25). In contrast to the increased interactions, the cross talk between lymphatic ECs and classical monocytes and alv macrophages via the *Icam1*-Integrin axis was decreased in the lung PMN of MMTV-PyVT mice. Similarly, communications between granulocytes and lymphatic ECs, alv macrophages and classical monocytes via *Ccl3*-based interactions were also reduced (Figure S16a, Table S26). Moreover, some interactions between lymphatic and capillary type I ECs in the lungs of wild-type mice were lost in the lungs of MMTV-PyVT mice, particularly those via the *Icam1*-*Itgav* and *Lrg1*-*Acvrl1* axis (Figure S16b and Table S27). The overview of the intercellular networks that were upregulated (Figure 7e) and downregulated are (Figure S6c) also shown.

### 3.7. The Role of S100A6 in EC Remodeling and Neutrophil Recruitment

**Figure 8:**
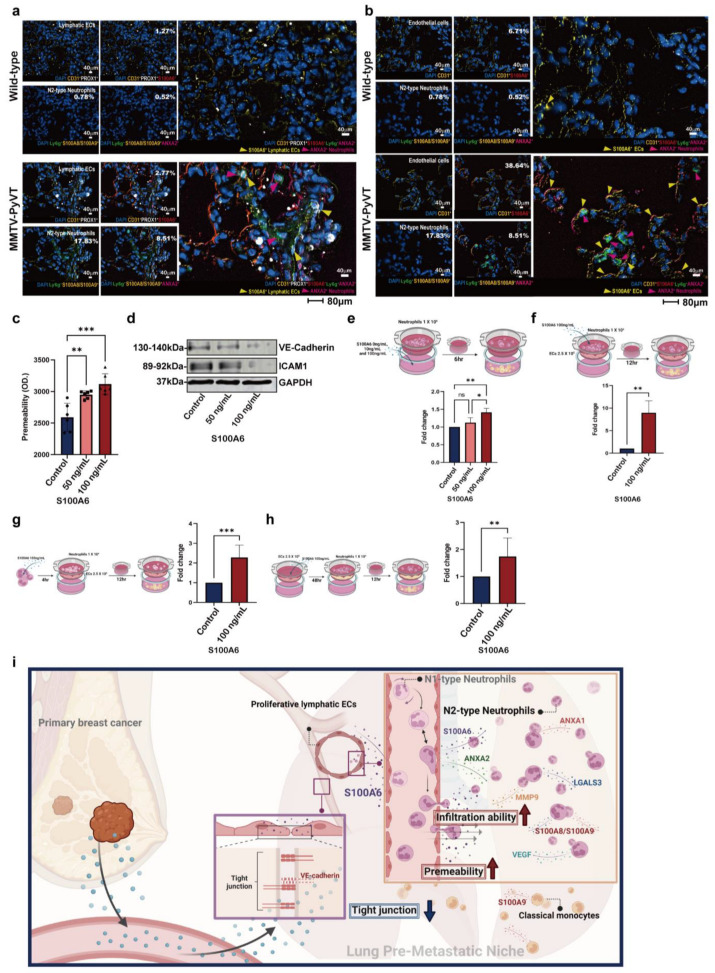
The roles of S100A6 in the vascular permeability and neutrophil recruitment. (**a**) The interaction between lymphatic ECs and N2-type neutrophils by S100A6-Anxa2 axis. The percentage of EC and neutrophil was indicated as *a percentage* of total CD31^+^ cell and total CD45^+^ cells, respectively. (**b**) Upregulated S100A6 expression in different cell types in the lungs of MMTV-PyVT mice. (**c**) S100A6 increased the vascular permeability of HULEC-5a. (**d**) S100A6 decreased the expression of tight junctions of HULEC-5a. (**e**) S100A6 cancer acted as a chemoattractant for migration. Neutrophils (1ꓫ10^6^ cells) were seeded in an insert and S100A6 protein added into the bottom. After 6 hr, the migratory neutrophils were counted. (**f**) S100A6 increased neutrophil transendothelial movement. HULEC-5a cells were seeded in an insert to form *confluent* monolayer, neutrophils (1ꓫ10^6^ cell) with or without S100A6 protein were added into the inserts. (**g**) S100A6 stimulated neutrophil migration. Neutrophil (1ꓫ10^6^ cell) were seeded in an insert (3 μm, 2.5 × 10^5^ cells) and then treated with S100A6 protein for 4 h after 12 h the migratory neutrophils were counted. (**h**) S100A6 increased transendothelial migration of neutrophils by changing ECs. HULEC-5a were seeded in an insert (3 μm, 2.5ꓫ10^5^ cells) and then treated with S100A6 protein for 48 h. After washing, the inserts were suspended in a 24-well plate and neutrophils (1 × 10^6^ cell) were added into the inserts for 12 h and migratory neutrophils were counted. (**i**) The proposed mechanism underlying the formation of the lung PMN in breast cancer. * *p*  <  0.05, ** *p*  <  0.01, *** *p* < 0.001.

The interaction of cells in the lungs of MMTV-PyVT mice was also validated by multiplex fluorescent staining. Neutrophils expressing higher levels of ANXA1 and LGALS3 were found in the lung PMN, but not in the lungs of wild-type mice (Figure S17a,b). Moreover, S100A6 expressing lymphatic ECs was surrounded by ANXA2 expressing N2-type neutrophils, verifying lymphatic EC-neutrophil interaction (Figure 8a and Figure S18a); additionally, the upregulation of S100A6 in vascular ECs was also observed in the lungs of MMTV-PyVT mice (Figure 8b and Figure S18b).

Several types of cells expressed higher levels of S100A6, which contributed to several interactions between different cell types in the lung PMN (Figure 7 and Figure S19), so functional assays of S100A6 in a cell model were further conducted. As shown in Figure 8e,f, S100A6 treatment decreased tight junctions VE-cadherin and ICAM1 expression and subsequently enhanced the permeability of human lung EC HULEC-5a (Figure 8c,d). S100A6 not only acted as a chemoattractant for neutrophil migration, but also stimulated neutrophil transendothelial migration in both pre-treatment and simultaneous treatment models (Figure 8e–g). Moreover, the transendothelial migration of neutrophils was increased in S100A6-pretreated HULEC-5a cells, consistent with the result of S100A6 decreasing tight junctions and increasing permeability of ECs (Figure 8h). These data showed that S100A6 mediated the remodeled endothelial cell and neutrophil recruitment in the lung PMN of mice with breast cancer.

## 4. Discussion

Metastasis is a pivotal determinant of clinical outcomes in patients with breast cancer [27], and the establishment of a PMN is considered to be a critical rate-limiting step during lung metastatic seeding in breast cancer [28,29]. In the current study, we demonstrated the important contributions of lymphatic ECs and N2-type neutrophils in the formation of the pulmonary PMN, and we elucidated the mechanisms underlying the interactions between local stromal and immune cells. Our study revealed that prior to metastasis, neutrophils have already been programmed to N2-type, as characterized by the upregulation of immunosuppressive factors. S100A6 can promote the recruitment of neutrophils to PMN by increasing the permeability of ECs and stimulating neutrophil movement, and with increased penetration of N2-type neutrophils, more circulating cancer cells might survive due to the immunosuppressive microenvironment. Our study established a mechanistic link between local ECs and neutrophils in the formation of a lung PMN for breast cancer metastasis (Figure 8i).

Numerous previous studies have indicated that neutrophils accumulate in the tumor microenvironment or metastatic niche to promote tumor metastasis [30,31], and that alveolar ECs are able to recruit neutrophils by secreting CXCL1, CXCL2, CXCL5 and CXCL12 after tumor-derived exosomal RNA stimulation [32]. Mesenchymal stromal cells can recruit neutrophils to the lung via C3 complement, and then stimulate NETosis in neutrophils [33]. Similar to the classification of M1/M2 macrophages, neutrophils are also divided into N1 and N2-types [34], with N2-type neutrophils being more similar to M2-type macrophages because of their immunosuppressive function, while N1-type neutrophils are superior at killing tumor cells [35,36,37]. N2-type neutrophils are now thought to contribute to the pathophysiology of various cancer metastasis and to also be a critical component of the PMN [26,38]. Our results showed that neutrophils infiltration in the lungs before metastasis is a critical feature of PMN, and their N2-type phenotype programming exhibits their ability to release several immunosuppressive factors including S100A8 and MMP9. In addition, NET-related genes, such as CD177 and LCN2, contributed to the phenotypic change of lung neutrophils in MMTV-PyVT mice; this supports the recently described elevated infiltration and activation of neutrophils, which contributes to various PMN formations and severe inflammatory pulmonary disorders [39,40,41,42]. In silico analysis of ligand–receptor interactions have revealed that neutrophils possess high ability to remodel classical monocytes, alv macrophages and alv fibroblasts, as well as ECs (capillary type I and lymphatic ECs), which were thought to undergo reprogramming during PMN formation [43,44]. Importantly, in vivo studies have indicated that neutrophils isolated from the lungs of MMTV-PyVT mice supported the theory that neutrophils were programmed to the N2-type phenotype and produced high levels of S100A8, MMP9 and VEGF, which were also demonstrated to play a critical role in the formation of the PMN [45,46]. These data suggest that neutrophils could be necessary for the early formation of lung PMNs, and that they have a significant influence on the immunity of the lung microenvironment in patients with breast cancer. The polarization of neutrophil to N2 phenotype and functional characterization in the blood of breast cancer patients could be further studied.

The involvement of the lung lymph system in the formation of PMNs in a specific distant organ is largely unknown. However, lymphatics and lymphatic ECs undergo conspicuous changes in idiopathic pulmonary fibrosis and other fibrotic lung diseases [47,48]. Lymphangiogenesis in the lung is increased by dendritic cells via the prostaglandin E2 receptor EP3-COX axis [49]. TNBC-derived IL6 induces CCL5 expression in stromal lymphatic vessels of the lung and lymph nodes through STAT3-AP1 signaling, resulting in tumor extravasation and colonization [50]. Our data, before lung metastasis in breast cancer, showed that lymphatic ECs increased in number concurrent with the recruitment of neutrophils in the lungs of mice. In silico analysis indicated that the PI3K signaling pathway was triggered, which is a well-known pathway for increasing cell proliferation, supported by the elevated levels of Ki67 in lung lymphatic ECs of MMTV-PyVT mice. Moreover, several cell types, including lymphatic ECs, secreted high levels of S100A6, which interrupted the tight VE-cadherin junctions of ECs, resulting in an increased level of transmembrane migration for neutrophils in our in vitro study. Our findings suggest that S100A6 released from proliferative lymphatic ECs attracts neutrophils and might trigger host pulmonary remodeling before lung metastasis of breast cancer.

The extensive information from scRNA-seq is a novel and powerful tool for providing knowledge of oncology and improving clinical diagnostics as well as drug development. Thus, the present study tried to find a novel mechanism and potential therapeutic markers for lung metastasis in breast cancer. However, the limitations of the present study should be noted. The first limitation of our study is the PMN change obtained from the lung of transgenic mice who drive transgene expression is not specified in the mammary ducts but also in several organs, including the lungs. Although we assessed the increase in neutrophils in both blood and lung and the change occurred at 10 weeks, it did not occur before 9 weeks, suggesting the enhancement and recruitment of neutrophil did not result from an effect of the polyoma virus middle T antigen. However, there is a possibility that the observed differences are certain to be due to the development of breast cancers in these mice and not to the expression of the transgene in the murine lungs. Other PMN features found in our study are required to be confirmed in the mice at different ages and more spontaneous lung metastasis of animal models is needed to validate the results in the results found in MMTV-PyVT transgene mice. Second, MMTV-PyVT breast cancer mouse mimic the human disease, however, some oncogenes vary between the two species and PyMT oncoprotein is not expressed in humans. Third, the DE gene in different cell subpopulations was performed according to the criteria (such as fold change and read counts). The results of pathway or functional analysis may be influenced by this subjective methodology and some key pathogenic factors may be ignored. Finally, our results are hard to reflect or prove by the lung PMN in humans due to ethical issues.

## 5. Conclusions

Our single-cell transcriptional analysis of the lungs of mice before breast cancer metastasis provides an insightful framework for understanding the establishment of the PMN and the cellular interaction patterns in the pulmonary environment. Mechanistically, lymphatic endothelium–neutrophil interactions, neutrophil N2-type phenotypic reprogramming and S100A6 upregulation in various lung host cells, further support inflammation-associated events which contribute to the formation of a lung PMN in mice with breast cancer. Collectively, our findings may provide a promising path towards the development of molecules targeted on neutrophils and lymphatic EC therapies for lung metastasis in breast cancer.

## Data Availability

The data that support the findings of this study are available from the corresponding author upon reasonable request.

## Supplementary Materials

The following supporting information can be downloaded at: https://www.mdpi.com/article/10.3390/cancers15010176/s1, Figure S1: Quality control of scRNA-seq, Figure S2: The cell clusters of monocyte/macrophage and dendritic cells, Figure S3: WGCNA analysis of different subsets of monocytes, Figure S4: Connective network of WGCNA modules (M1 and M6 modules) for monocytes, Figure S5: KEGG pathways of (a) M1 and (b) M4 modules, Figure S6: Trajectory analysis of monocytes, Figure S7: Trajectory analysis of granulocytes, Figure S8: WGCNA analysis of N1- and N2-type neutrophils, Figure S9: Connective network of modules for N1- and N2-type neutrophil WGCNA (M 1 to M10 modules) analysis, Figure S10: The KEGG pathways of (a) M 4, (b) M 8, (c) M 5 and (d) M 10 modules, Figure S11: Trajectory analysis of ECs, Figure S12: WGCNA analysis of lymphatic ECs, Figure S13: The networks of modules in lymphatic endothelial cells, Figure S14: WGCNA analysis of stroma cells, Figure S15: The network of WCGNA modules of stroma cells, Figure S16: Downregulated cell-cell interactions of the cell types of the lungs, Figure S17: The secretions of Anax1 (a) and galectin-3 (LGALS3) (b) was found in the N2-type neutrophils (CD45+Ly6g+S100A8/A9+) of the lung PMN, Figure S18: The interactions of ECs with neutrophils, Figure S19: The enhanced levels of S100A6 in several cell types of lungs in MMTV-PyVT mice; Table S1: Cell markers, Table S2: Trajectory analysis of non-classical and classical monocytes, Table S3: WGCNA of non-classical and classical monocytes, Table S4: KEGG pathways of M4 module of monocytes, Table S5: KEGG pathways of M1 module of monocytes, Table S6: Trajectory analysis of N1- and N2-type neutrophils, Table S7: WGCNA of N1- and N2-type neutrophils, Table S8: KEGG pathways of M4 module of N1- and N2-type neutrophils, Table S9: KEGG pathways of M5 module of N1- and N2-type neutrophils, Table S10: KEGG pathways of M7 module of N1- and N2-type neutrophils, Table S11: KEGG pathways of M8 module of N1- and N2-type neutrophils, Table S12: KEGG pathways of M10 module of N1- and N2-type neutrophil, Table S13: Canonical pathways of lymphatic ECs assessed by IPA, Table S14: WGCNA of ECs, Table S15: KEGG pathways of M1 module of lymphatic ECs, Table S16: KEGG pathways of M2 module of lymphatic ECs, Table S17: IPA analysis of alv fibroblasts, Table S18: IPA analysis of adv fibroblasts, Table S19: WCGNA of fibroblasts, Table S20: KEGG pathways of M2 module of fibroblasts, Table S21: KEGG pathways of M3 module of fibroblasts, Table S22: KEGG pathways of M4 module of fibroblasts, Table S23: KEGG pathways of M5 module of fibroblasts, Table S24: Cell-cell interactions only in the lungs of MMTV-PyVT mice, Table S25: Elevated cell-cell interactions in the lungs of MMTV-PyVT mice, Table S26: Reduced cell-cell interactions in the lungs of MMTV-PyVT mice, Table S27: Loss of cell-cell interactions in the lungs of MMTV-PyVT mice, Table S28: The primers used in this study, Table S29: The antibodies used in study.
