# Supplementary material for: Single-Cell Transcriptomic Profiles of Lung Pre-Metastatic Niche Reveal Neutrophil and Lymphatic Endothelial Cell Roles in Breast Cancer
Source: Cancers (Basel). 2022 Dec 28;15(1):176. doi: 10.3390/cancers15010176 (PMC9818165; doi:10.3390/cancers15010176)
Supplement: Supplementary file 1 [file cancers-15-00176-s001.zip › Supplementary figure legens.pdf]

Supplementary data

Figure S1. Quality control of scRNA-seq. (a) knee plot of single cell RNA-seq profile (b) Batch-effect correction. (c) Scatterplot illustrating the number of genes, unique molecular identifiers (UMIs) and the percentage of mitochondrial genes in each cell of three lung samples.

Figure S2. The cell clusters of monocyte/macrophage and dendritic cells. (a) The blueprint of myeloid cells. (b) The trajectory analysis of myeloid cells. The phenotypic changes of myeloid cells, as determine by the Monocle 2 algorithm.

Figure S3. WGCNA analysis of different subsets of monocytes. (a) The correlation between modules and different types of monocytes. The top 25 hub genes in (b) M1, (c) M 2 and (d) M 4 modules.

Figure S4. Connective network of WGCNA modules (M1 and M6 modules) for monocytes.

Figure S5. KEGG pathways of (a) M1 and (b) M4 modules.

Figure S6. Trajectory analysis of monocytes. (a) Genes that involved in the phenotypic change. (b) The genes contributed to non-classical and classical monocytes.

Figure S7. Trajectory analysis of granulocytes. (a) Trajectory analysis of granulocytes and neutrophils. (b) The genes contributed the phenotypic change of N1- and N2-type neutrophils.

Figure S8. WGCNA analysis of N1- and N2-type neutrophils. (a) WGCNA analysis (b) The correlation of modules in N1- and N2-type neutrophils. The top 25 hub genes in eigengene network of (c) M4, (d) M 5, (e) M 7, (f) M 8, (g) and M 10 modules.

Figure S9. Connective network of modules for N1- and N2-type neutrophil WGCNA (M 1 to M10 modules) analysis.

Figure S10. The KEGG pathways of (a) M 4, (b) M 8, (c) M 5 and (d) M 10 modules.

Figure S11. Trajectory analysis of ECs. (a) Trajectory analysis for endothelial cells. (b) The Venn diagram of genes of all EC clusters. *(*c) Expression of NRAS and PIK3CA in lymphatic ECs.

Figure S12. WGCNA analysis of lymphatic ECs. (a) WGCNA cluster dendrogram and module assignment. The colors in the horizontal bar represented the modules. The eigengene network of (b) M 1 and (c) M 2 modules.

Figure S13. The networks of modules in lymphatic endothelial cells.

Figure S14. WGCNA analysis of stroma cells. The eigengene networks of (a) M 1, (b) M 2, (c) M 3, (d) M 4, and (e) M 5 modules.

Figure S15. The network of WCGNA modules of stroma cells.

Figure S16. Downregulated cell-cell interactions of the cell types of the lungs. (a) Interactions between lymphatic ECs and granulocytes. (b) The cross talk between capillary type I ECs with lymphatic ECs. (c) The total network of downregulated cell-

cell communication in the lung PMN.

Figure S17. The secretions of Anax1 (a) and galectin-3 (LGALS3) (b) was found in the N2-type neutrophils ($CD45^{+}Ly6g^{+}S100A8/A9^{+}$) of the lung PMN.

Figure S18. The interactions of ECs with neutrophils. (a) S100A6 involved in the interaction lymphatic ECs with N2-type neutrophils. (b) The cell-cell communication of ECs with neutrophils by S100A6.

Figure S19. The enhanced levels of S100A6 in several cell types of lungs in MMTV-PyVT mice.
