# Supplementary material for: Single-Cell Transcriptomic Profiles of Lung Pre-Metastatic Niche Reveal Neutrophil and Lymphatic Endothelial Cell Roles in Breast Cancer
Source: Cancers (Basel). 2022 Dec 28;15(1):176. doi: 10.3390/cancers15010176 (PMC9818165; doi:10.3390/cancers15010176)
Supplement: Supplementary file 1 [file cancers-15-00176-s001.zip › Supplementary Table legens.pdf]

Supplementary data

Table S1. Cell markers

Table S2. Trajectory analysis of non-classical and classical monocytes

Table S3. WGCNA of non-classical and classical monocytes

Tabe S4. KEGG pathways of M4 module of monocytes

Tabe S5. KEGG pathways of M1 module of monocytes

Table S6. Trajectory analysis of N1- and N2-type neutrophils

Table S7. WGCNA of N1- and N2-type neutrophils

Table S8. KEGG pathways of M4 module of N1- and N2-type neutrophils

Table S9. KEGG pathways of M5 module of N1- and N2-type neutrophils

Table S10. KEGG pathways of M7 module of N1- and N2-type neutrophils

Table S11. KEGG pathways of M8 module of N1- and N2-type neutrophils

Table S12. KEGG pathways of M10 module of N1- and N2-type neutrophil

Table S13. Canonical pathways of lymphatic ECs assessed by IPA

Table S14. WGCNA of ECs

Table S15. KEGG pathways of M1 module of lymphatic ECs

Table S16. KEGG pathways of M2 module of lymphatic ECs

Table S17. IPA analysis of alv fibroblasts

Table S18. IPA analysis of adv fibroblasts

Table S19. WCGNA of fibroblasts

Table S20. KEGG pathways of M2 module of fibroblasts

Table S21. KEGG pathways of M3 module of fibroblasts

Table S22. KEGG pathways of M4 module of fibroblasts

Table S23. KEGG pathways of M5 module of fibroblasts

Table S24. Cell-cell interactions only in the lungs of MMTV-PyVT mice

Table S25. Elevated cell-cell interactions in the lungs of MMTV-PyVT mice

Table S26. Reduced cell-cell interactions in the lungs of MMTV-PyVT mice

Table S27. Loss of cell-cell interactions in the lungs of MMTV-PyVT mice

Table S28. The primers used in this study

Table S29. The antibodies used in study
