# Supplementary material for: Single-Cell Transcriptomic Profiles of Lung Pre-Metastatic Niche Reveal Neutrophil and Lymphatic Endothelial Cell Roles in Breast Cancer
Source: Cancers (Basel). 2022 Dec 28;15(1):176. doi: 10.3390/cancers15010176 (PMC9818165; doi:10.3390/cancers15010176)
Supplement: Supplementary file 1 [file cancers-15-00176-s001.zip › Table S28.pdf]

Table S28. The primers used in this study

Gene	Primer sequence	Note
Ccl3	Forward: 5’-ccaagtcttctcagcgccat-3’	
	Reverse: 5’-TTGGAGTCAGCGCAGATCTG-3’	
Arg1	Forward: 5’-caacactcccctgacaacca-3’	
	Reverse: 5’-CTCGCAAGCCAATGTACACG-3’	
Mmp9	Forward: 5’-agctggcagaggcatacttg-3’	
	Reverse: 5’-GGCCTTTAGTGTCTGGCTGT-3’	
Icam1	Forward: 5’-ccactgccttggtagaggtg-3’	
	Reverse: 5’-TACTTGGCTCCCTTCCGAGA-3’	
Actb	Forward: 5’-tacccaggcattgctgacag-3’	
	Reverse: 5’-ACTCCTGCTTGCTGATCCAC-3’	
olMR8744	Forward: 5’-CAAATGTTGCTTGTCTGGTG-3’	Internal Positive Control Forward
olMR8745	Reverse: 5’-GTCAGTCGAGTGCACAGTTT-3’	Internal Positive Control Reverse
olMR0384	Forward: 5’-GGAAGCAAGTACTTCACAAGGG-3’	Transgene Forward
olMR0385	Reverse: 5’-GGAAAGTCACTAGGAGCAGGG-3’	Transgene Reverse
