# Supplementary material for: Single-Cell Transcriptomic Profiles of Lung Pre-Metastatic Niche Reveal Neutrophil and Lymphatic Endothelial Cell Roles in Breast Cancer
Source: Cancers (Basel). 2022 Dec 28;15(1):176. doi: 10.3390/cancers15010176 (PMC9818165; doi:10.3390/cancers15010176)
Supplement: Supplementary file 1 [file cancers-15-00176-s001.zip › Table S29.pdf]

Table S29. The antibodies used in study

Antibodies for flow cytometry					
Antibody	Fluorophore	Clone	Vander	Catalog Number	Dilutions
Ly-6G	PE	1A8	BD Pharmingen	551461	1:200
CD11b	APC	M1/70	BD Pharmingen	553312	3:200
CD45	APC	30-F11	BD Pharmingen	559864	1:100
Ly-6G	PE/Cyanine7	1A8	BD Pharmingen	560601	2.5:200
Arginase 1/ARG1	PE	Polyclonal	R&D Systems	IC5868P	3:100
PE Rat IgG2a, κ Isotype Control	PE	R35-95	BD Pharmingen	553930	1:200
APC Rat IgG2b, κ Isotype Control	APC	A95-1	BD Pharmingen	553991	3:200/1:100
PE-Cy™7 Mouse IgG2a, κ Isotype Control	PE/Cyanine7	G155-178	BD Pharmingen	557907	2.5:200
Sheep IgG PE-conjugated Antibody	PE	Polyclonal	R&D Systems	IC016P	3:100
Antibody for western blot					
Antibody	Host	Clone	Vander	Catalog Number	Dilutions
VE-Cadherin	Rabbit	D87F2	Cell Signaling	2500	1:1000
CD54 (ICAM1)	Rabbit	E3Q9N	Cell Signaling	67836	1:1000
GAPDH	Mouse	6C5	Merck Millipore	MAB374	1:5000
Antibody for multi-color immunohistochemistry					
Antibody	Host	Clone	Vander	Catalog Number	Dilutions
Annexin A2 (ANXA2)	Rabbit	D11G2	Cell Signaling	8235	1:50
Annexin A1 (ANXA1)	Rabbit	EPR19342	abcam	ab222398	1:200
S100 alpha 6 (S100A)	Rabbit	EPR13084-69	abcam	ab181975	1:100
PROX1	Rabbit	Polyclonal	abcam	Ab101851	1:200
S100A8 + S100A9	Rabbit	RM1038	abcam	Ab288578	1:500
Galectin 3 (LGALS3)	Rabbit	A3A12	abcam	Ab2785	1:250
CCR2	Mouse	3G7	Novus Biologicals	NBP2-35334	1:100
Endothelin R Type B (EDNRB)	Rabbit	Polyclonal	Novus Biologicals	NBP1-31108	1:200
CD31	-	MEC13.3	Akoya Biosciences	4250001	1:200
CD45	-	30-F11	Akoya Biosciences	4150002	1:200
Ly6G	-	1A8	Akoya Biosciences	4350015	1:200
CD11b	-	M1/70	Akoya Biosciences	4150015	1:200
Ki67	-	B56	Akoya Biosciences	4250019	1:200
