# Supplementary figures and images for: Single-Cell Transcriptomic Profiles of Lung Pre-Metastatic Niche Reveal Neutrophil and Lymphatic Endothelial Cell Roles in Breast Cancer
Source: Cancers (Basel). 2022 Dec 28;15(1):176. doi: 10.3390/cancers15010176 (PMC9818165; doi:10.3390/cancers15010176)

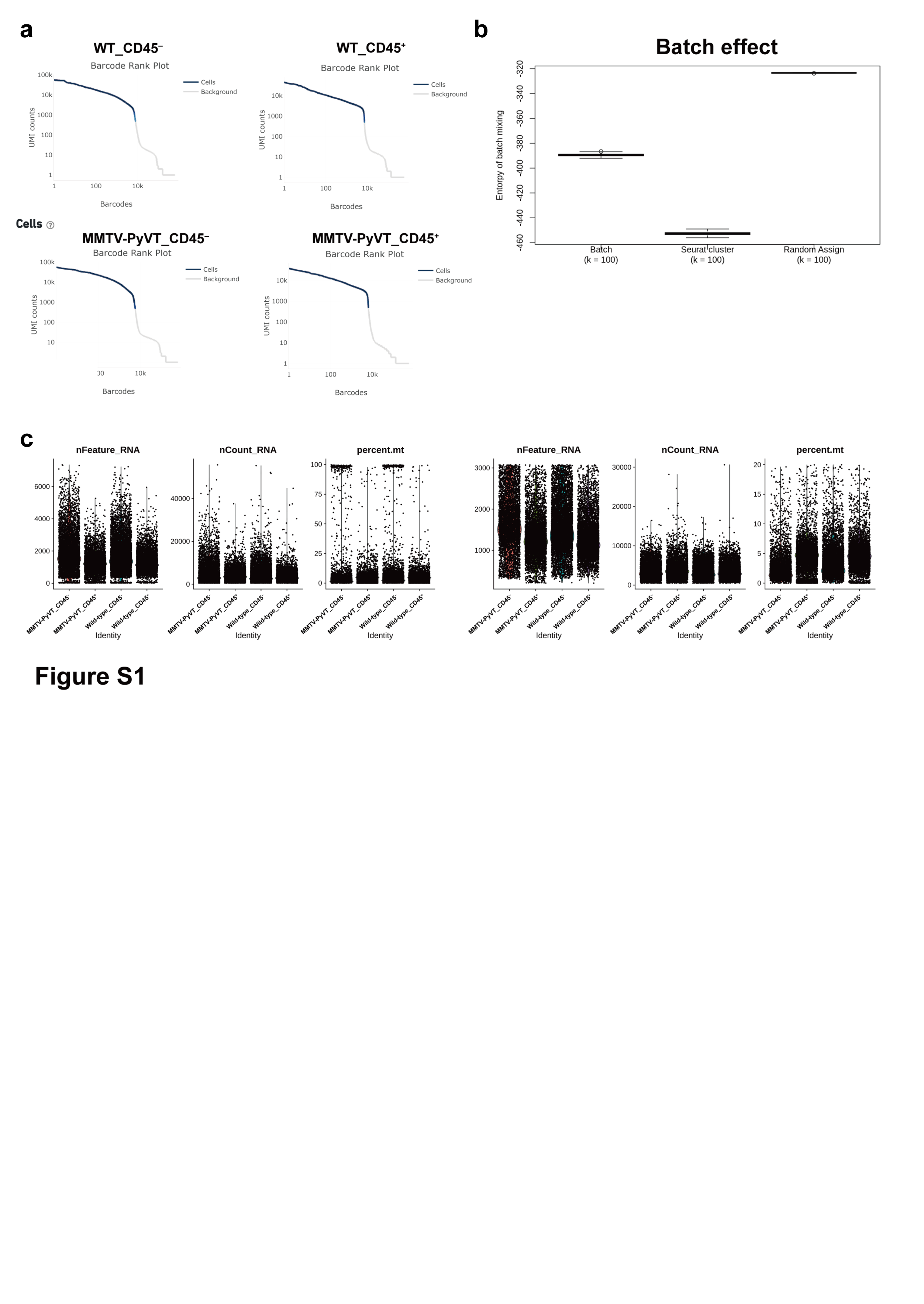

Supplement: Supplementary file 1 [file cancers-15-00176-s001.zip › Figure S1.tif]

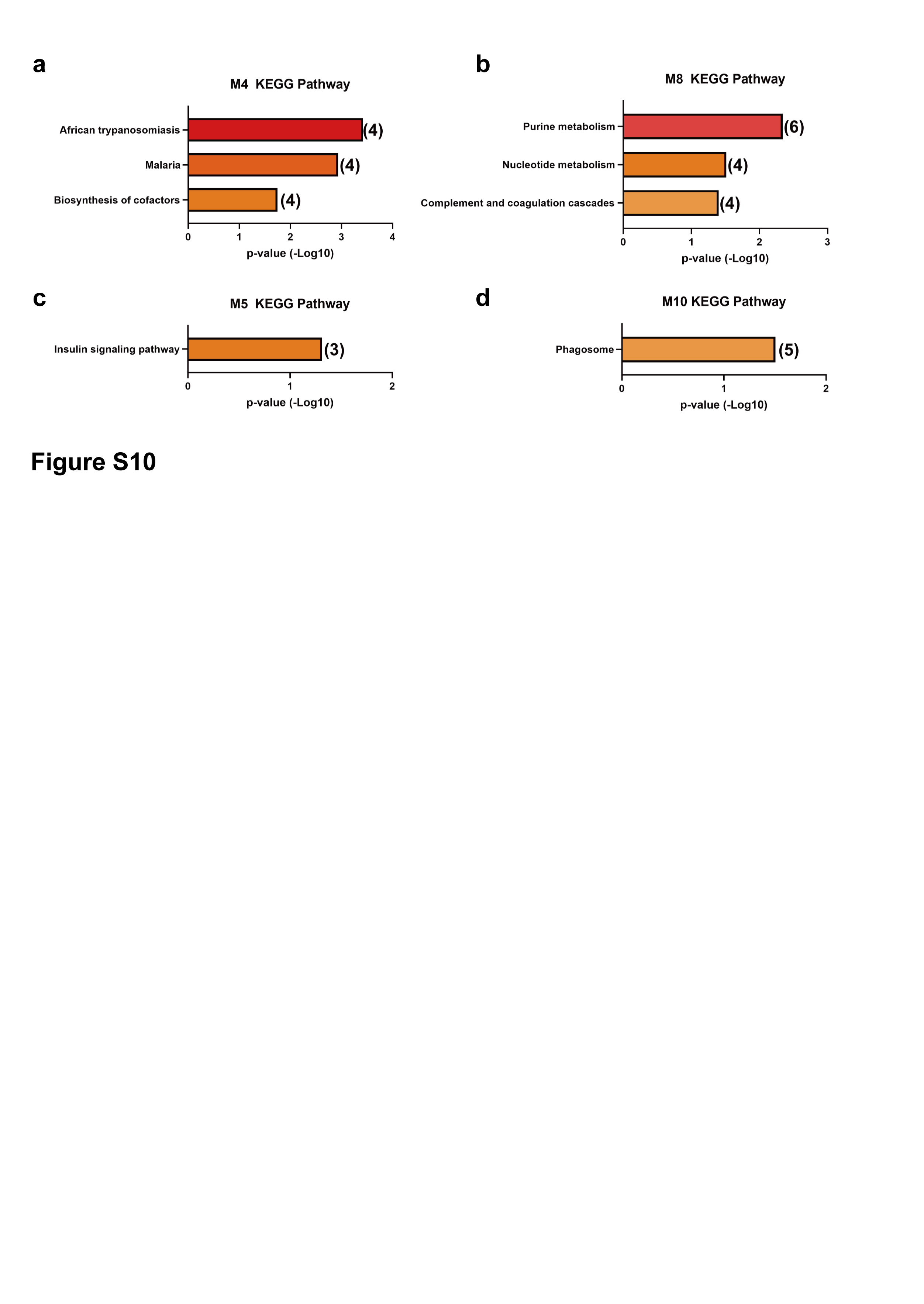

Supplement: Supplementary file 1 [file cancers-15-00176-s001.zip › Figure S10.tif]

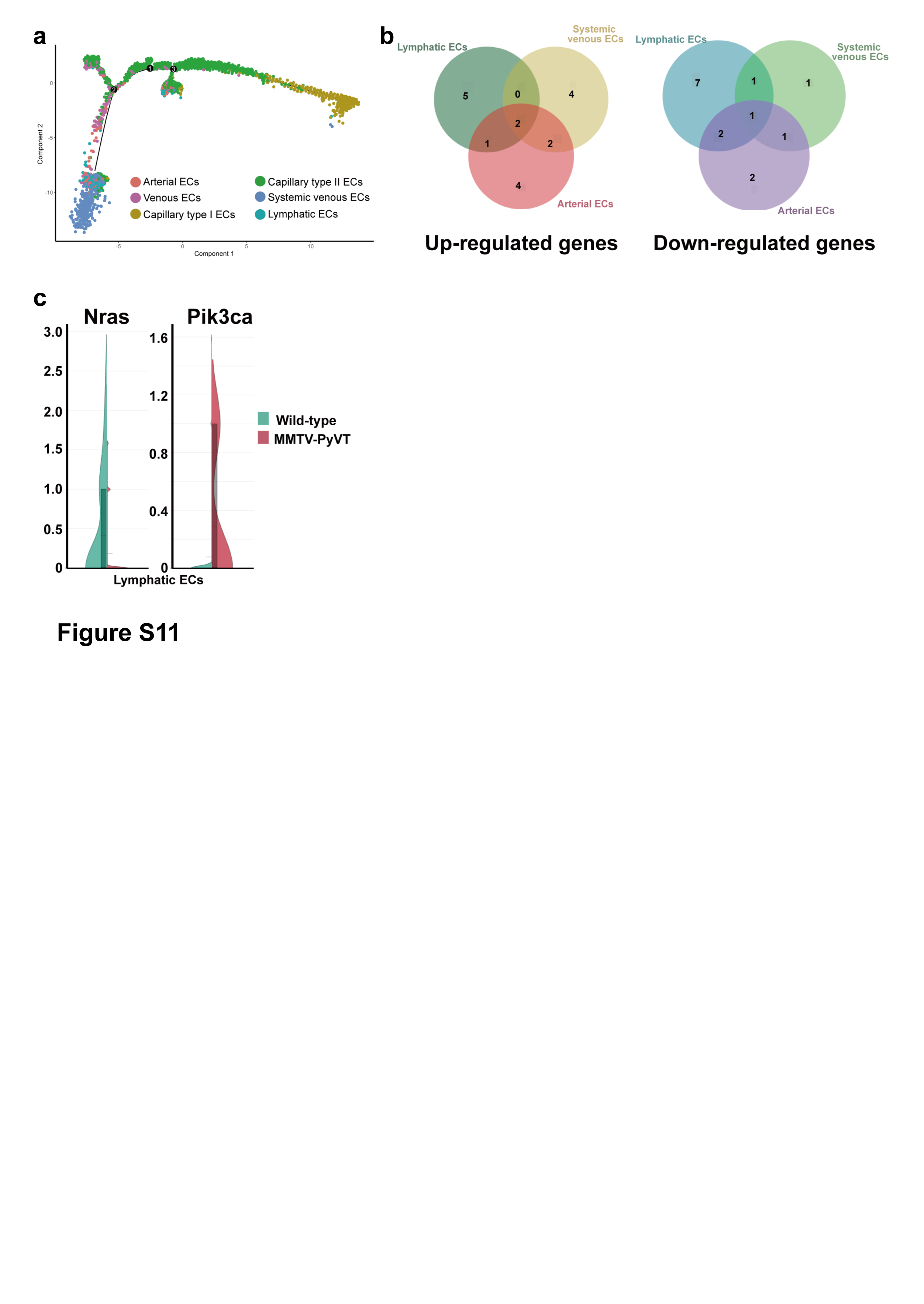

Supplement: Supplementary file 1 [file cancers-15-00176-s001.zip › Figure S11.tif]

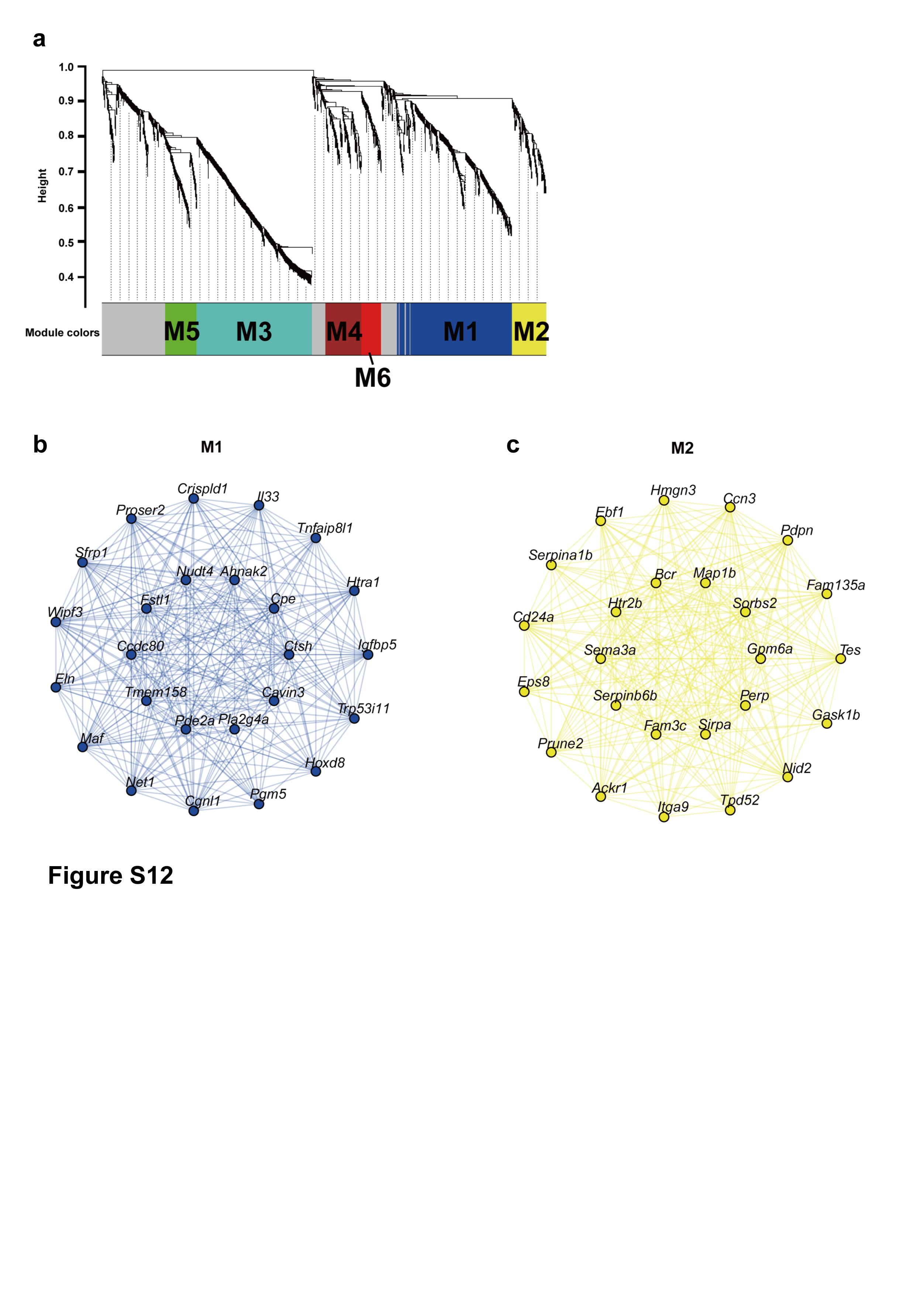

Supplement: Supplementary file 1 [file cancers-15-00176-s001.zip › Figure S12.tif]

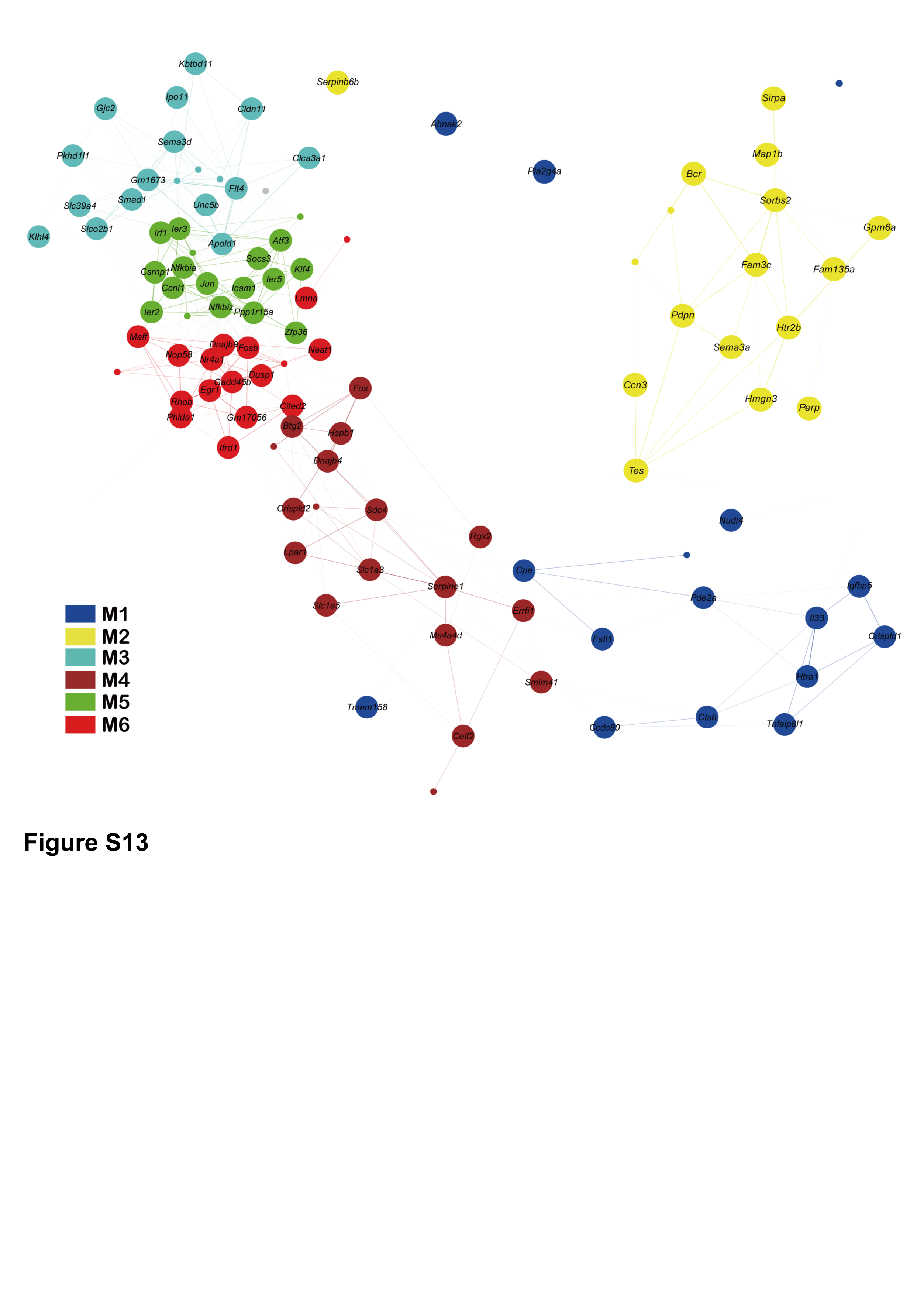

Supplement: Supplementary file 1 [file cancers-15-00176-s001.zip › Figure S13.tif]

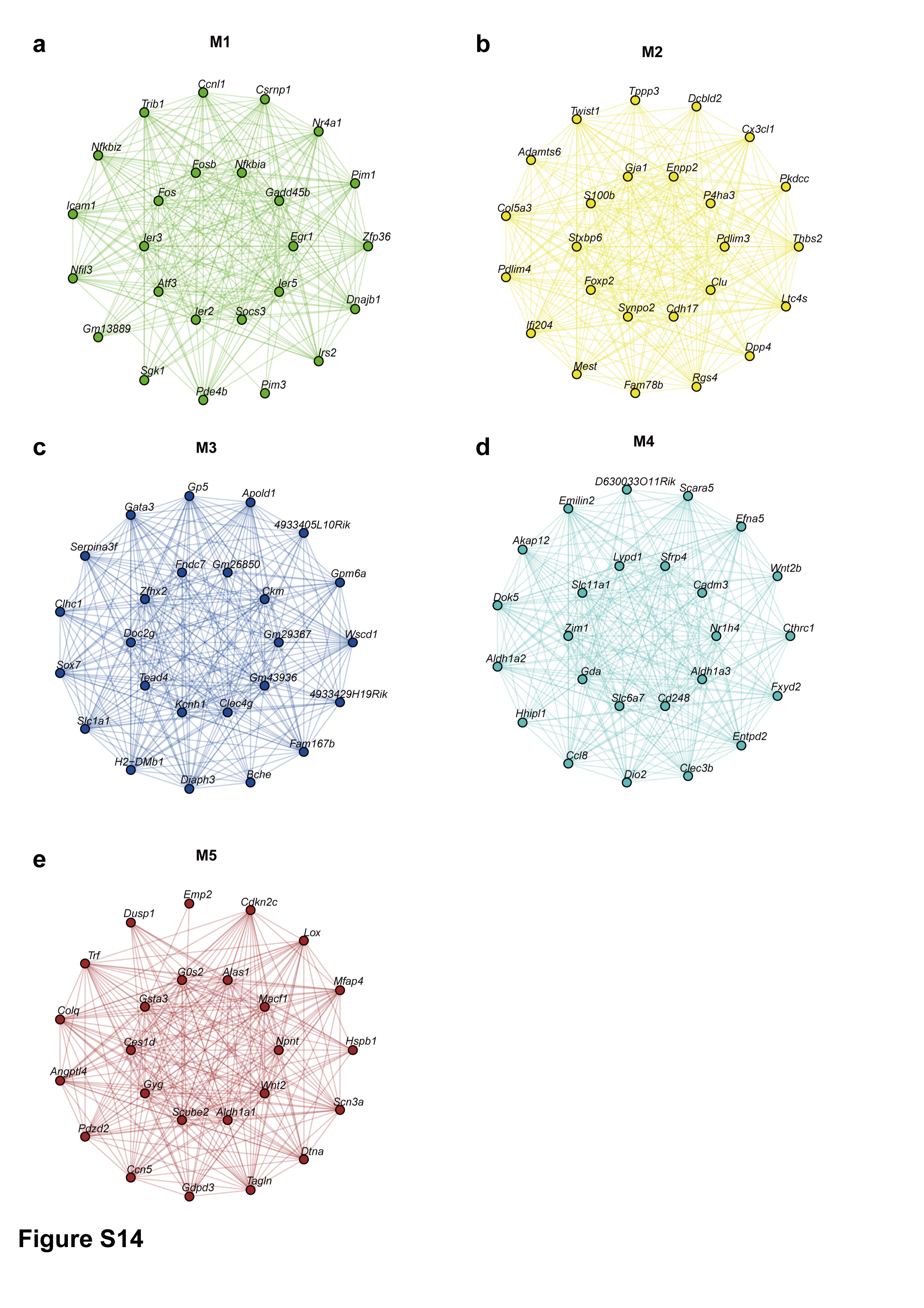

Supplement: Supplementary file 1 [file cancers-15-00176-s001.zip › Figure S14.tif]

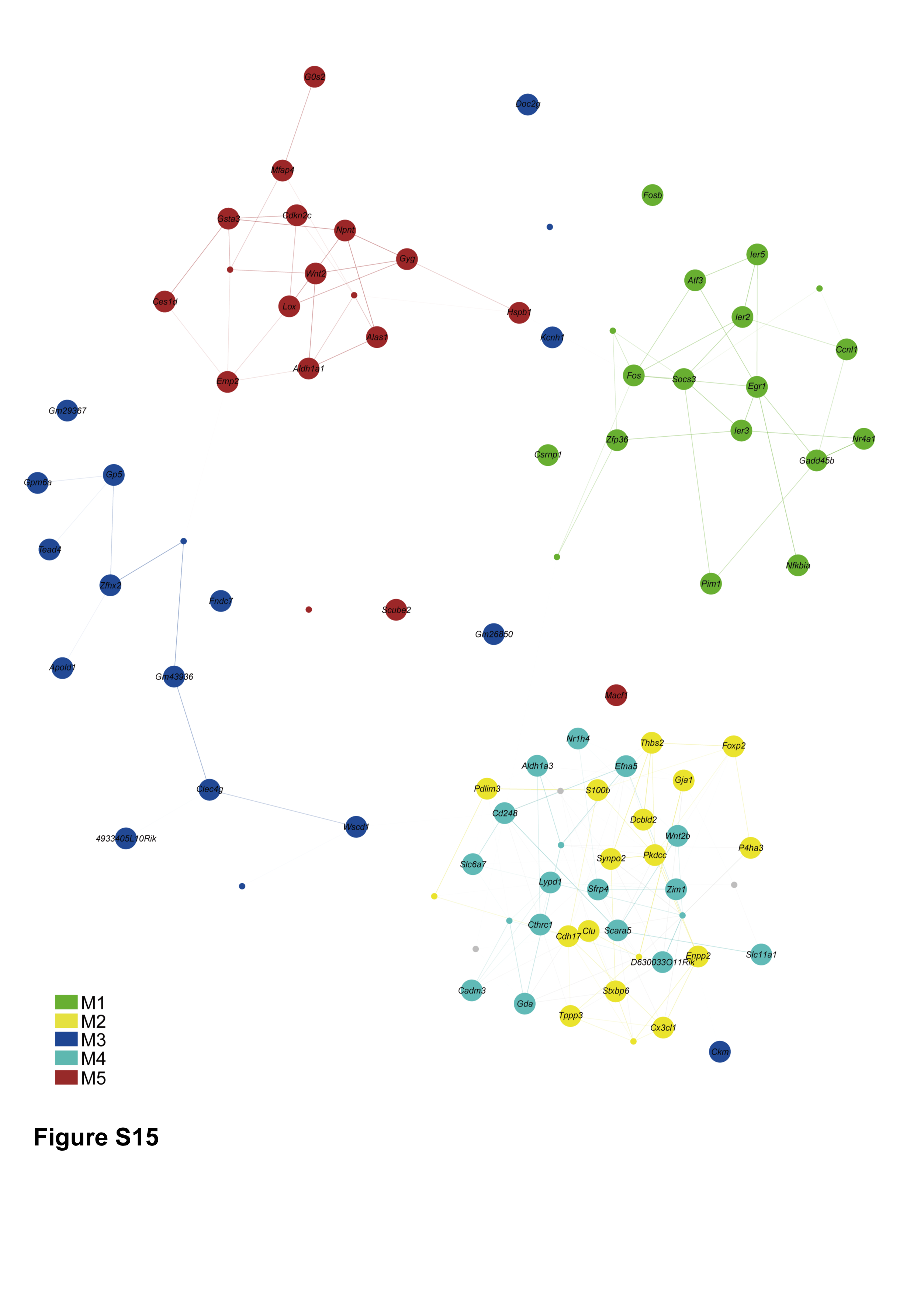

Supplement: Supplementary file 1 [file cancers-15-00176-s001.zip › Figure S15.tif]

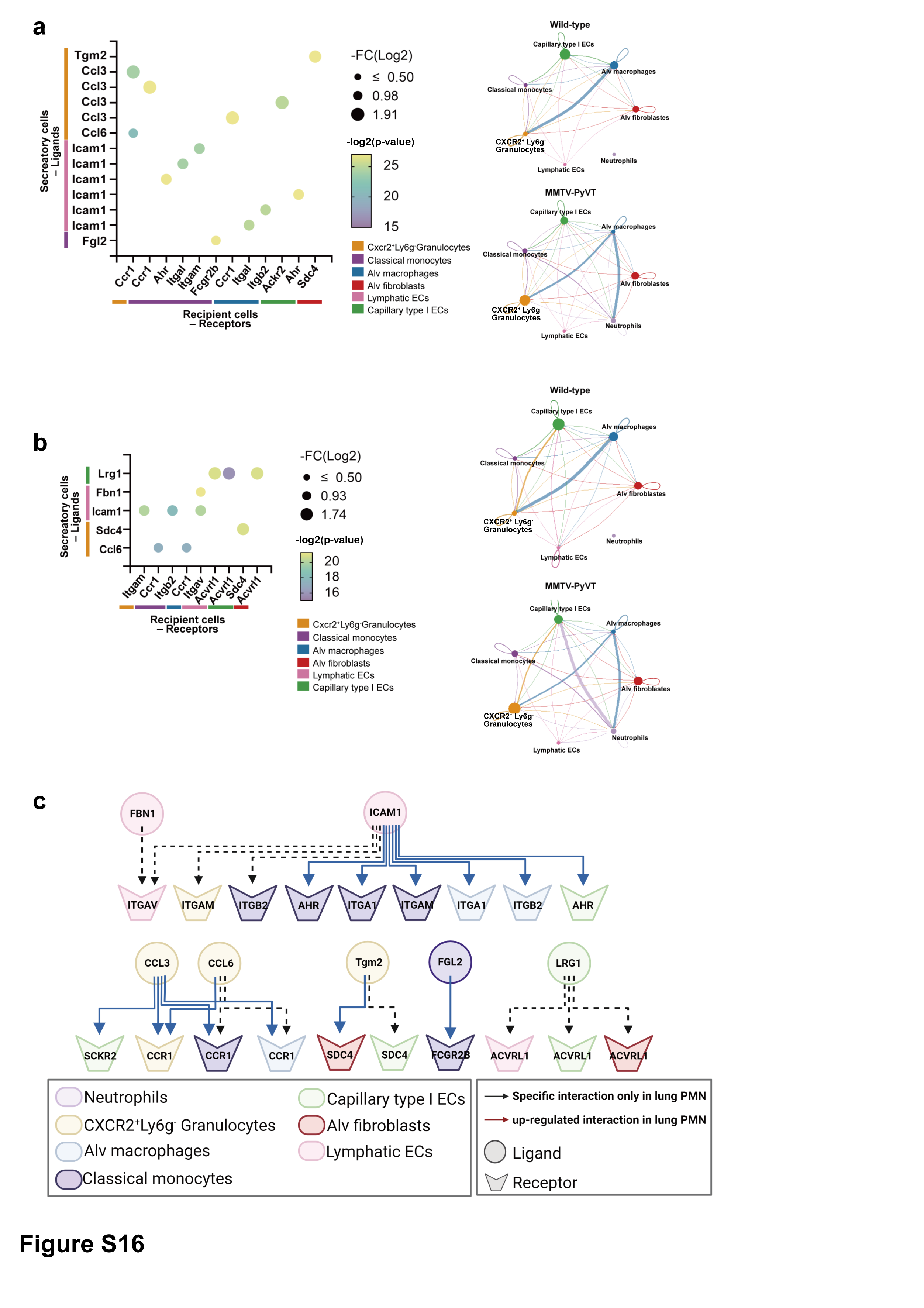

Supplement: Supplementary file 1 [file cancers-15-00176-s001.zip › Figure S16.tif]

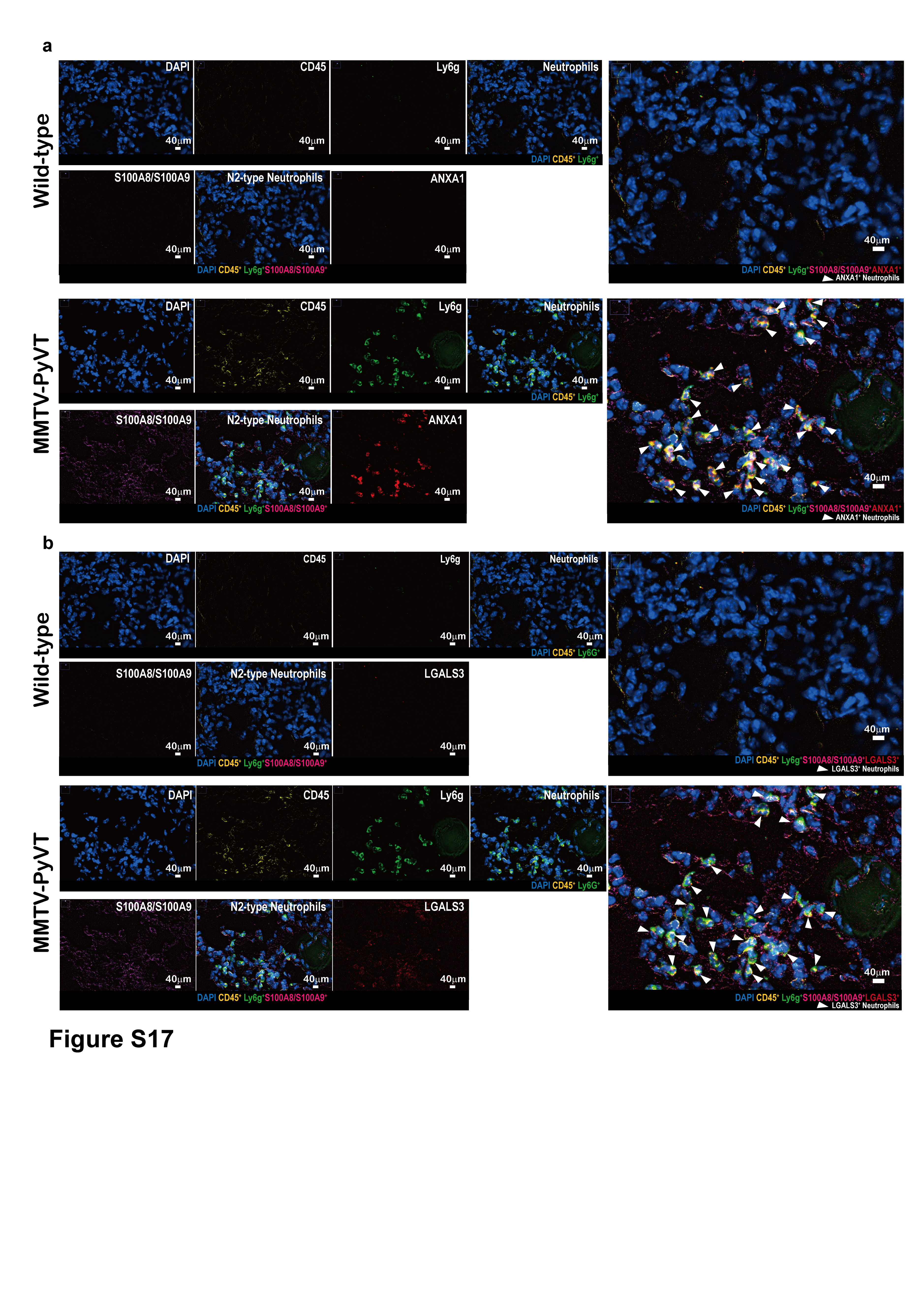

Supplement: Supplementary file 1 [file cancers-15-00176-s001.zip › Figure S17.tif]

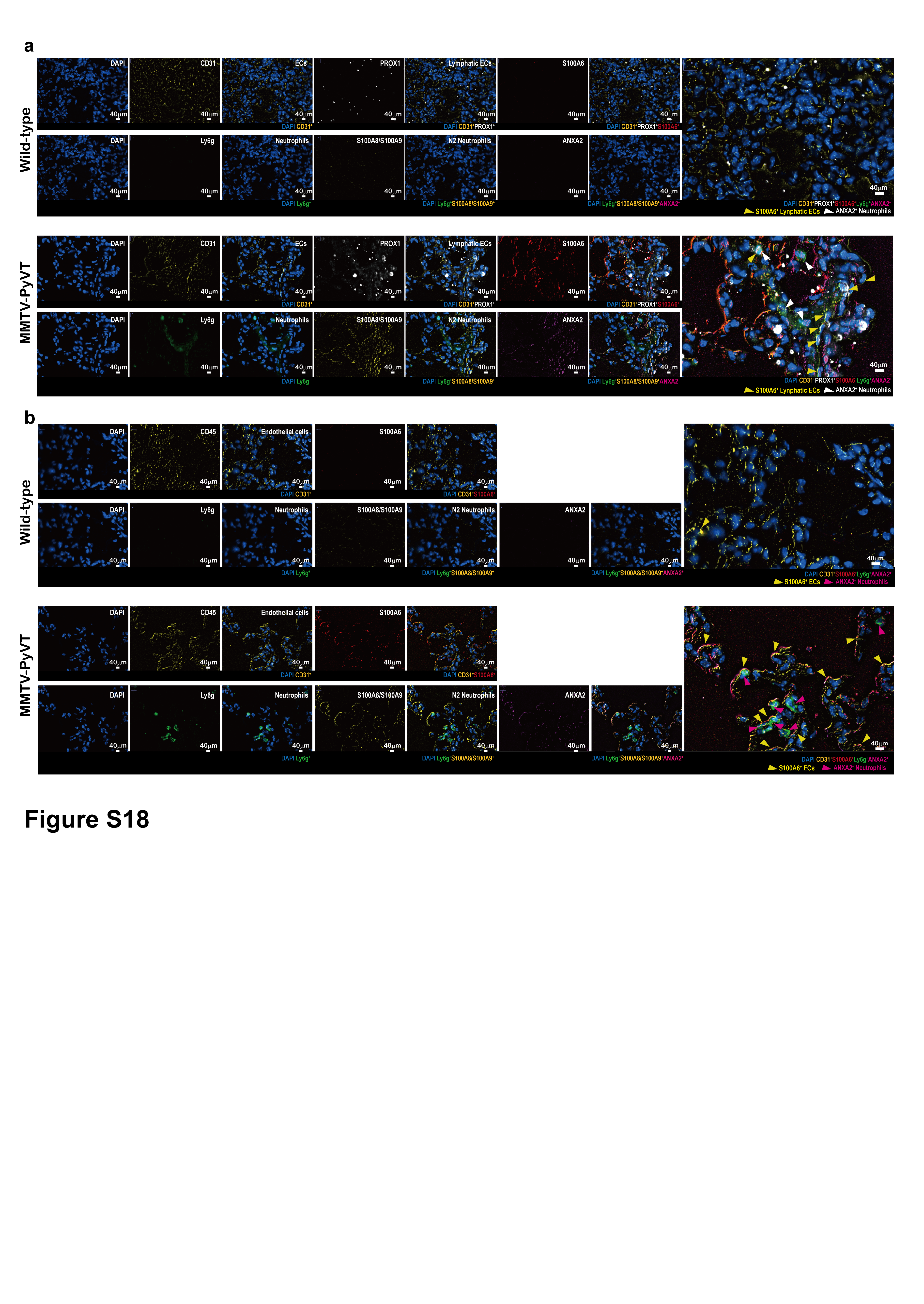

Supplement: Supplementary file 1 [file cancers-15-00176-s001.zip › Figure S18.tif]

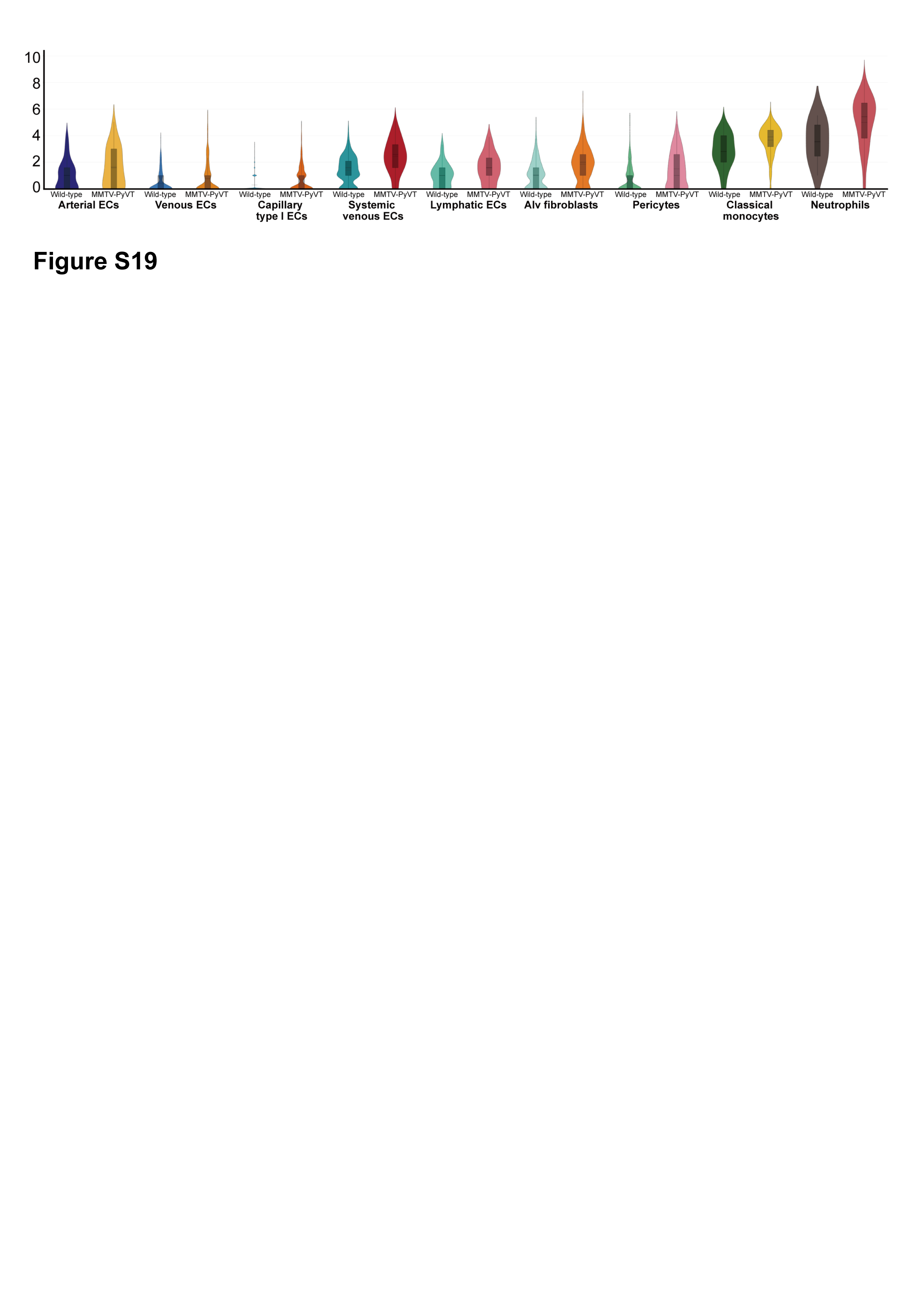

Supplement: Supplementary file 1 [file cancers-15-00176-s001.zip › Figure S19.tif]

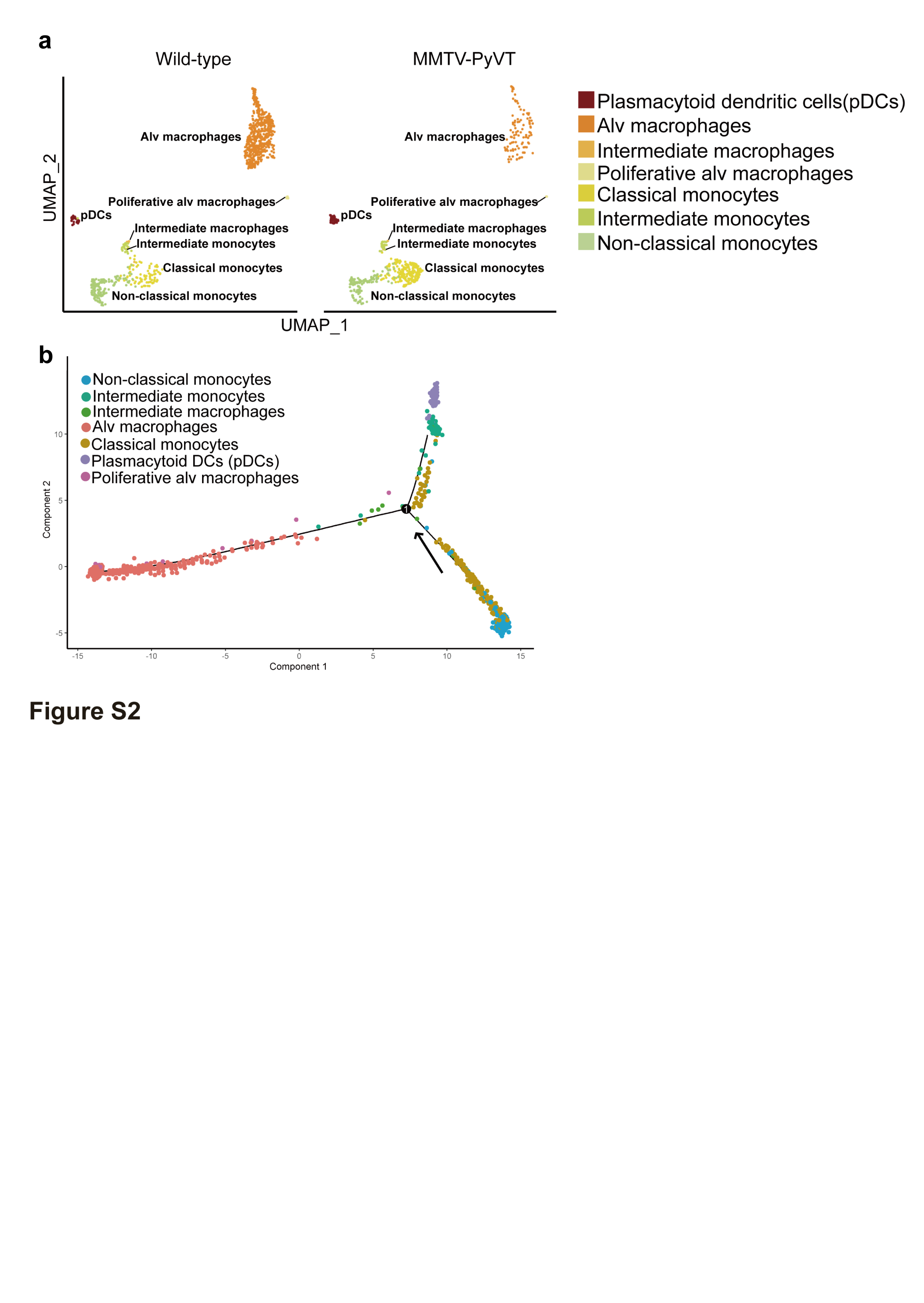

Supplement: Supplementary file 1 [file cancers-15-00176-s001.zip › Figure S2.tif]

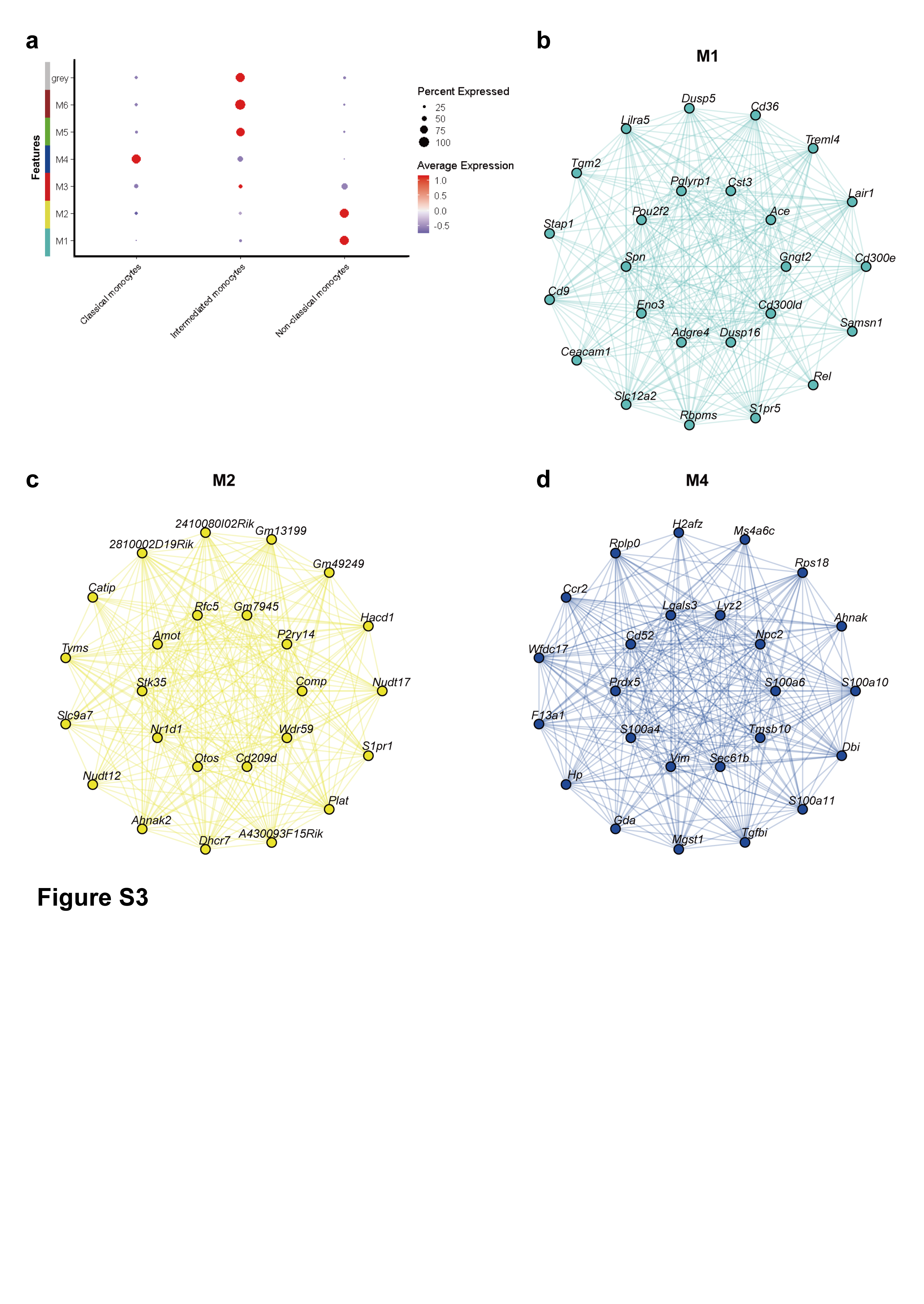

Supplement: Supplementary file 1 [file cancers-15-00176-s001.zip › Figure S3.tif]

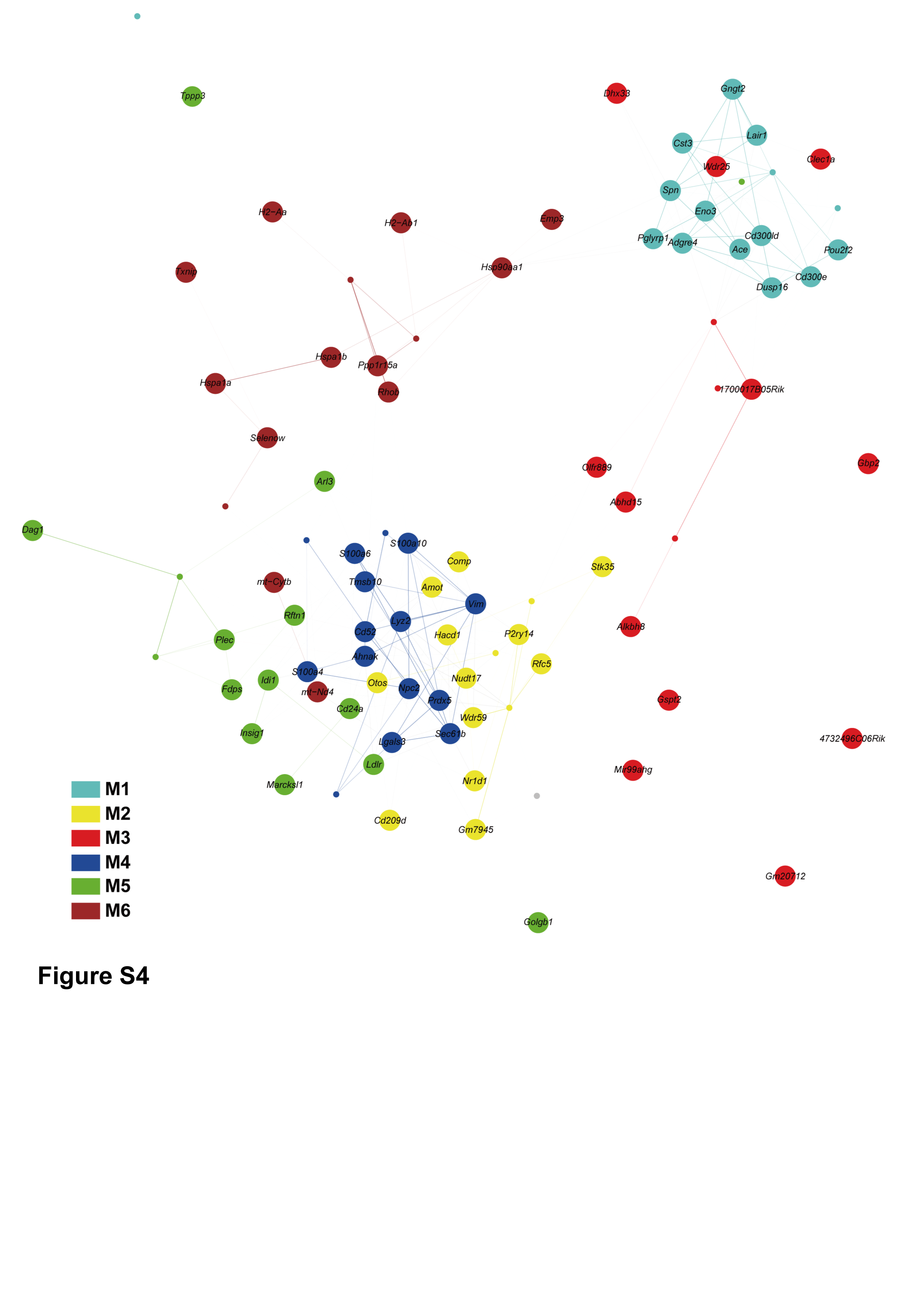

Supplement: Supplementary file 1 [file cancers-15-00176-s001.zip › Figure S4.tif]

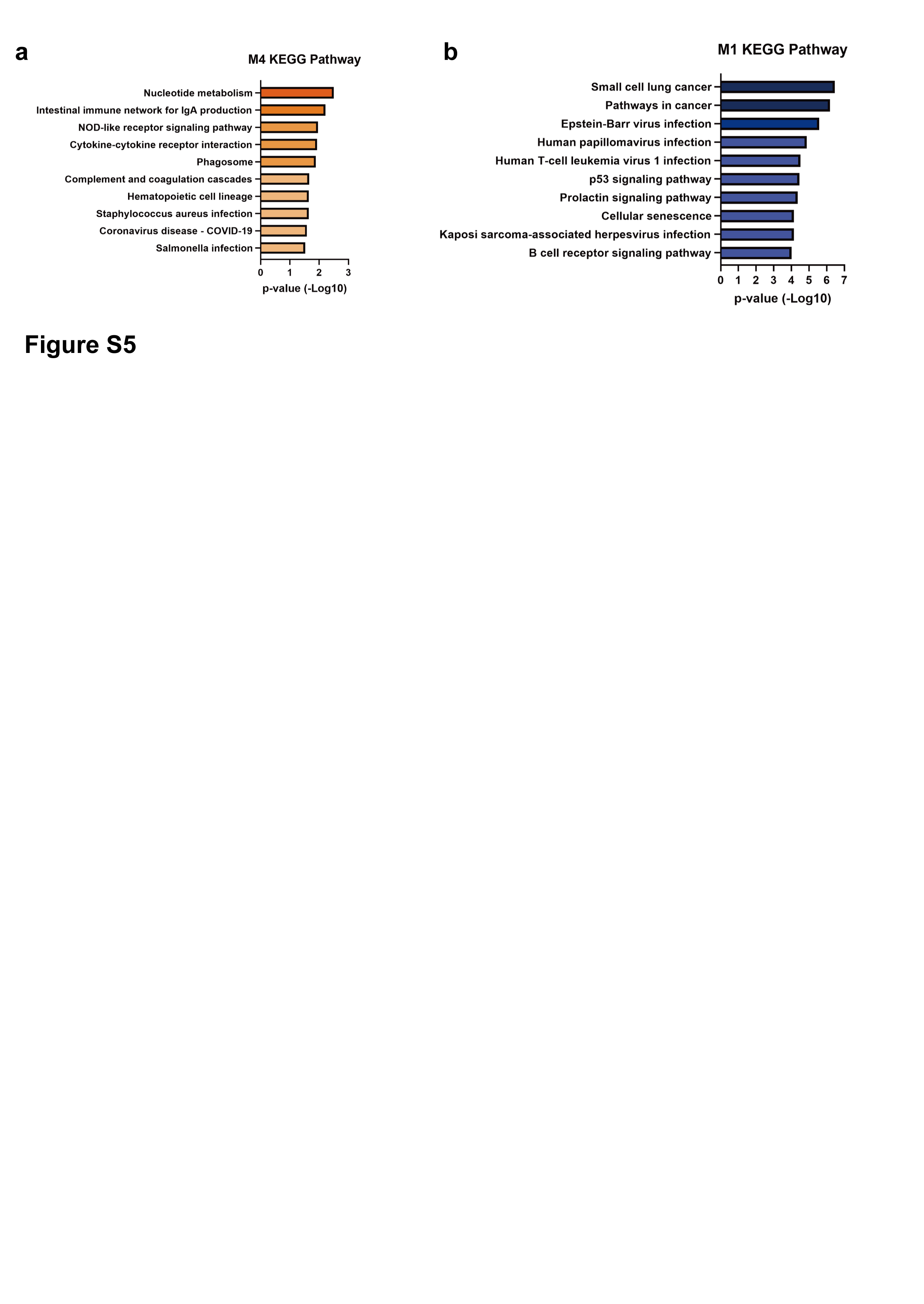

Supplement: Supplementary file 1 [file cancers-15-00176-s001.zip › Figure S5.tif]

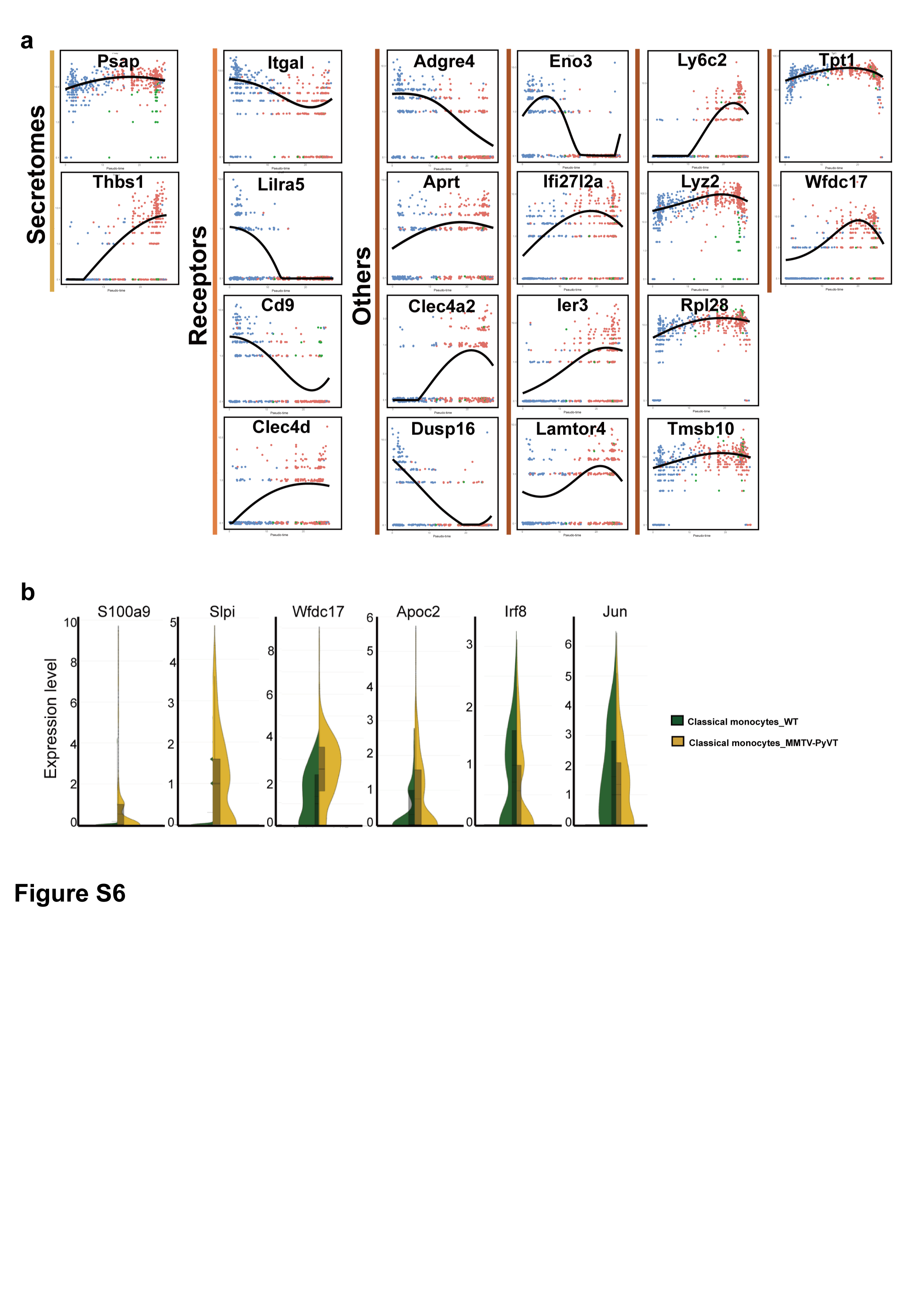

Supplement: Supplementary file 1 [file cancers-15-00176-s001.zip › Figure S6.tif]

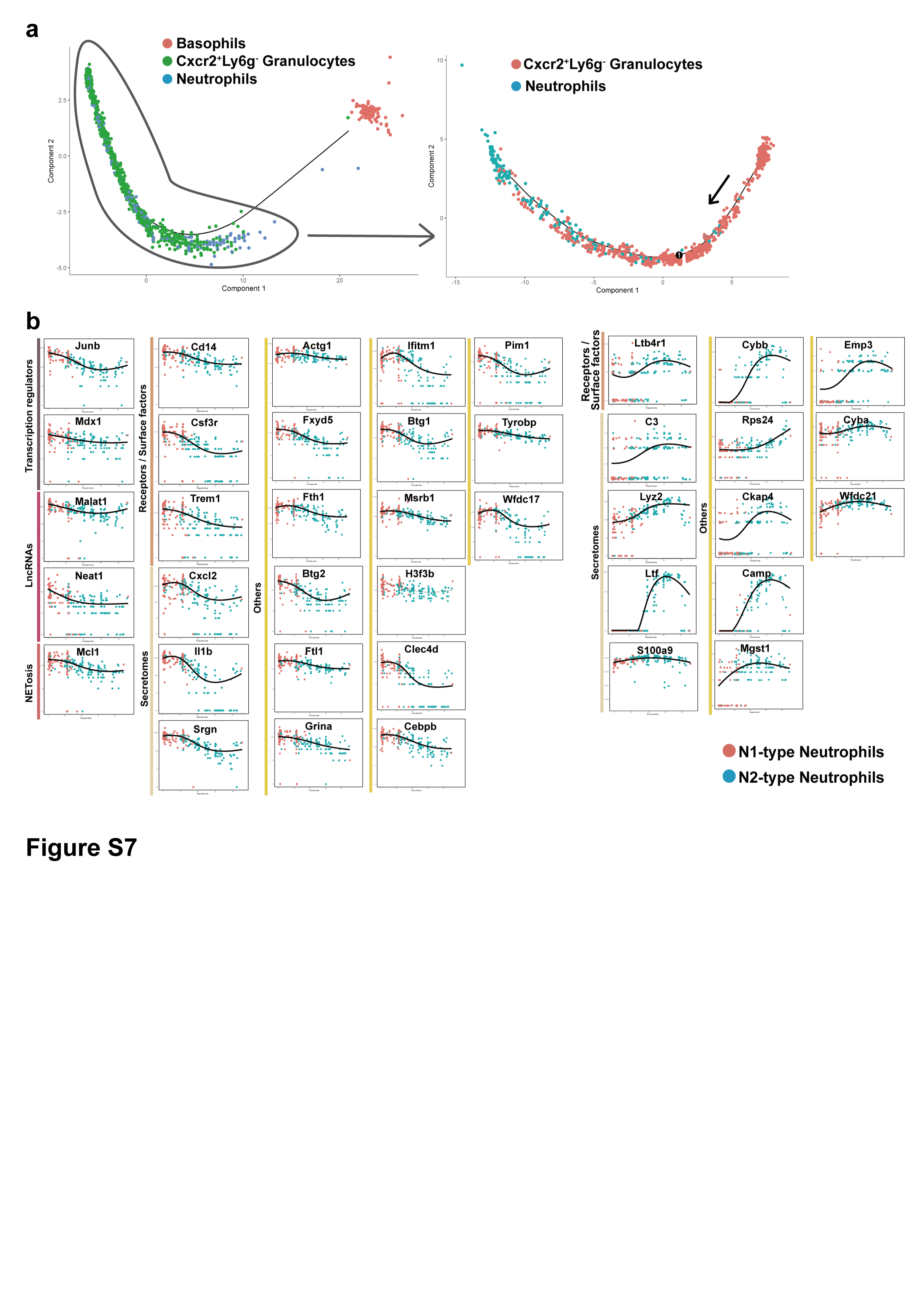

Supplement: Supplementary file 1 [file cancers-15-00176-s001.zip › Figure S7.tif]

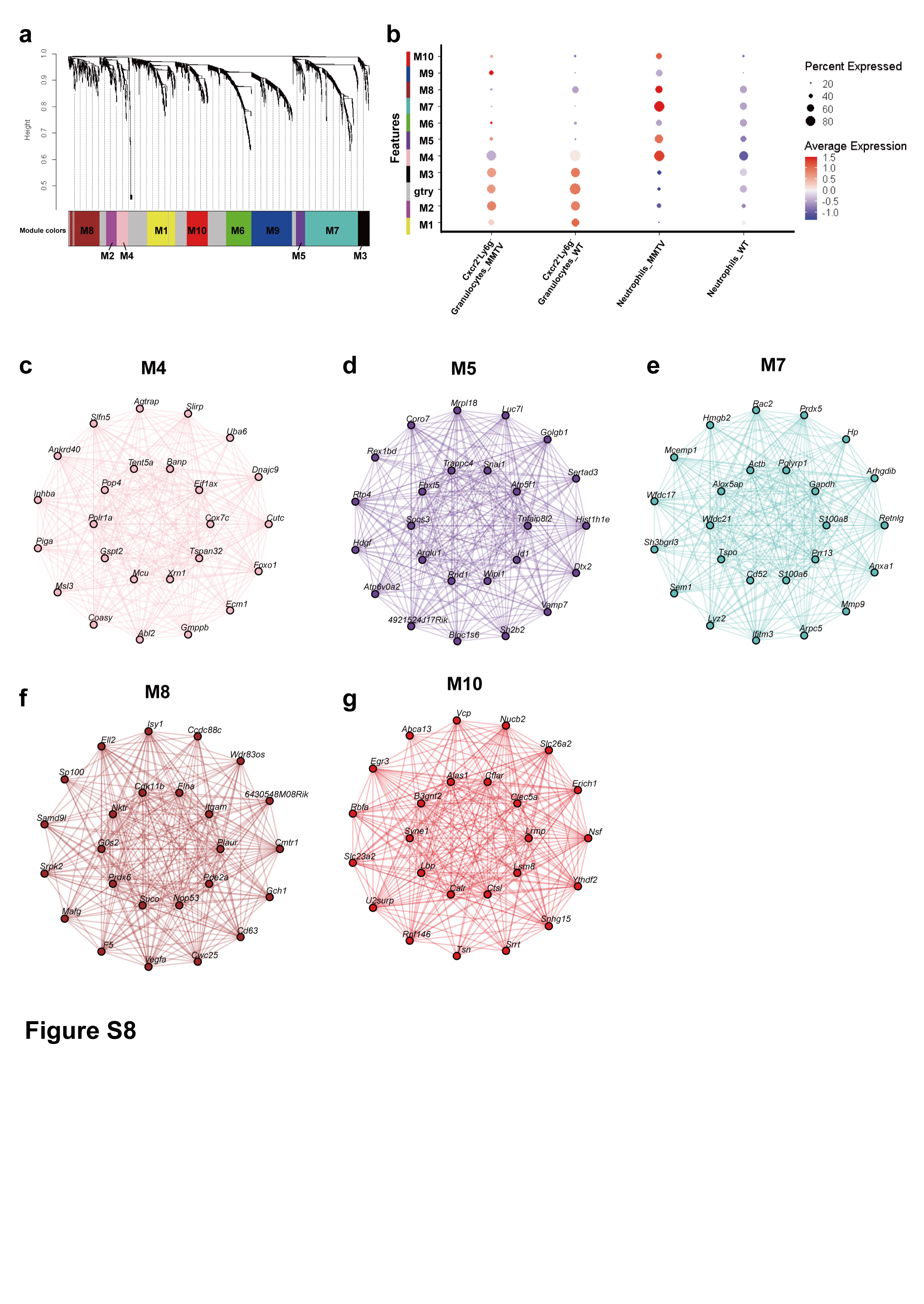

Supplement: Supplementary file 1 [file cancers-15-00176-s001.zip › Figure S8.tif]

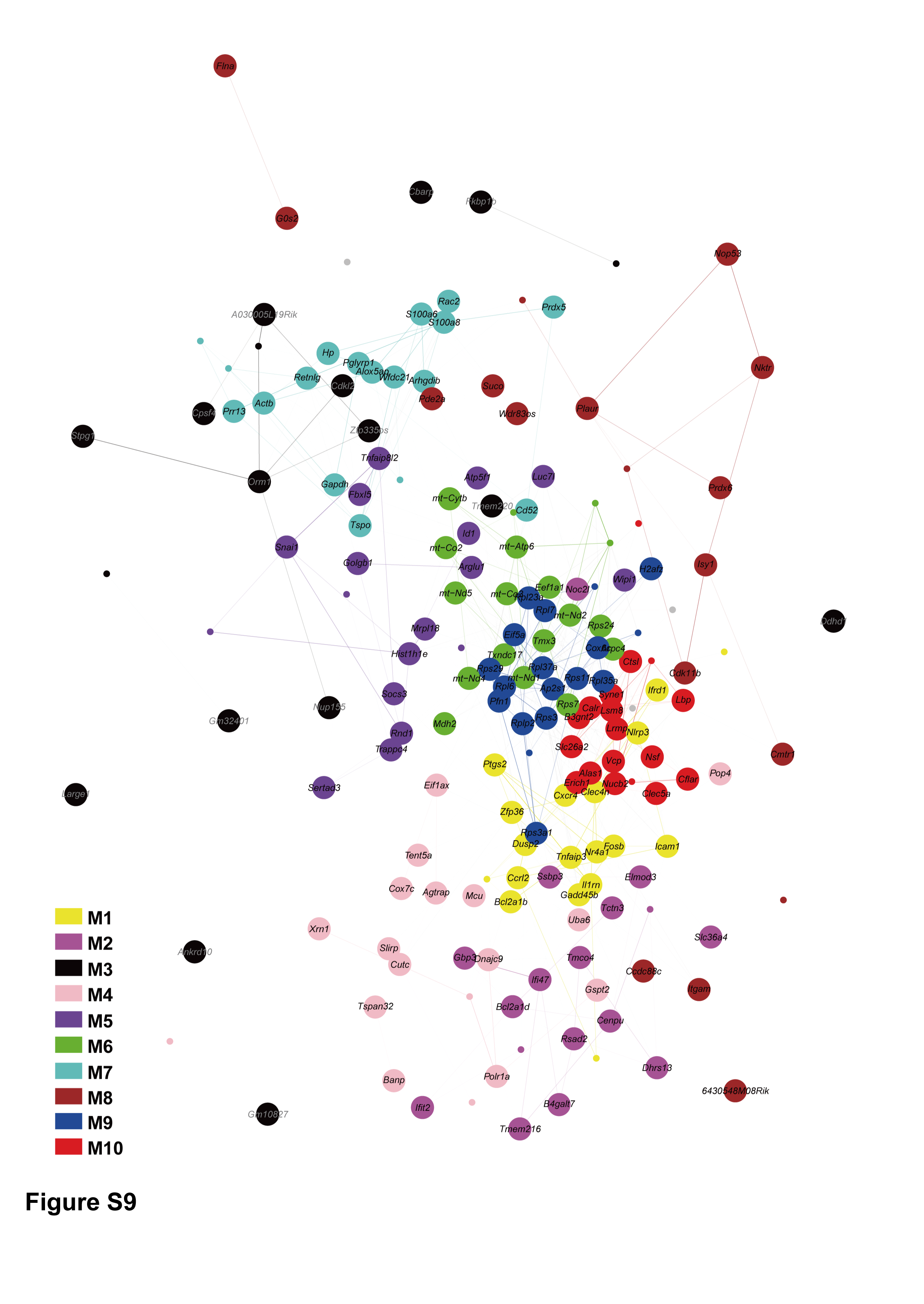

Supplement: Supplementary file 1 [file cancers-15-00176-s001.zip › Figure S9.tif]
